# A novel cyclic γ-AApeptide-based long-acting pan-coronavirus fusion inhibitor with potential oral bioavailability by targeting two sites in spike protein

**DOI:** 10.1038/s41421-022-00455-6

**Published:** 2022-09-08

**Authors:** Songyi Xue, Xinling Wang, Lei Wang, Wei Xu, Shuai Xia, Lujia Sun, Shaohui Wang, Ning Shen, Ziqi Yang, Bo Huang, Sihao Li, Chuanhai Cao, Laurent Calcul, Xingmin Sun, Lu Lu, Jianfeng Cai, Shibo Jiang

**Affiliations:** 1 Department of Chemistry, University of South Florida, 4202 E Fowler Ave., Tampa, FL USA; 2 Key Laboratory of Medical Molecular Virology (MOE/NHC/CAMS), Shanghai Institute of Infectious Disease and Biosecurity, School of Basic Medical Sciences, Shanghai Frontiers Science Center of Pathogenic Microbes and Infection, Fudan University, Shanghai, China; 3 Department of Molecular Medicine, Morsani College of Medicine, University of South Florida, Tampa, FL USA; 4 Department of Pharmaceutical Science, Taneja College of Pharmacy, University of South Florida, Tampa, FL USA

**Keywords:** Molecular modelling, Mechanisms of disease

## Abstract

The receptor-binding domain (RBD) in S1 subunit and heptad repeat 1 (HR1) domain in S2 subunit of SARS-CoV-2 spike (S) protein are the targets of neutralizing antibodies (nAbs) and pan-coronavirus (CoV) fusion inhibitory peptides, respectively. However, neither nAb- nor peptide-based drugs can be used orally. In this study, we screened a one-bead-two-compound (OBTC) cyclic γ-AApeptide library against SARS-CoV-2 S protein and identified a hit: **S-20** with potent membrane fusion inhibitory activity, but moderate selectivity index (SI). After modification, one derivative, **S-20-1**, exhibited improved fusion inhibitory activity and SI (>1000). **S-20-1** could effectively inhibit infection by pseudotyped and authentic SARS-CoV-2 and pseudotyped variants of concern (VOCs), including B.1.617.2 (Delta) and B.1.1.529 (Omicron), as well as MERS-CoV, SARS-CoV, HCoV-OC43, HCoV-229E, and HCoV-NL63. It could also inhibit infection of a pseudotyped SARS-related coronavirus WIV1 (SARSr-CoV-WIV1) from bats. Intranasal application of **S-20-1** to mice before or after challenge with HCoV-OC43 or SARS-CoV-2 provided significant protection from infection. Importantly, **S-20-1** was highly resistant to proteolytic degradation, had long half-life, and possessed favorable oral bioavailability. Mechanistic studies suggest that **S-20-1** binds with high affinity to RBD in S1 and HR1 domain in S2 of SARS-CoV-2 S protein. Thus, with its pan-CoV fusion and entry inhibitory activity by targeting two sites in S protein, desirable half-life, and promising oral bioavailability, **S-20-1** is a potential candidate for further development as a novel therapeutic and prophylactic drug against infection by SARS-CoV-2 and its variants, as well as future emerging and reemerging CoVs.

## Introduction

Several vaccines^1^ and therapeutics^2^ have been approved for use against COVID-19 caused by SARS-CoV-2 infection. However, their effectiveness against the emerging variants of SARS-CoV-2, such as the B.1.1.7 (Alpha)^3^, B.1.351 (Beta)^4^, B.1.1.248 (Gamma)^5^, B.1.617.2 (Delta)^6^, and B.1.1.529 (Omicron)^7^ appear to decline. Therefore, developing more effective and broader spectrum prophylactics and therapeutics is still urgently needed.

Coronaviruses consist of four genera: Alphacoronavirus (α), Betacoronavirus (β), Gammacoronavirus (γ) and Deltacoronavirus (δ)^7^. Two Alphacoronaviruses (HCoV-NL63 and HCoV-229E) and 5 Betacoronaviruses, including low pathogenic CoVs (HCoV-OC43, HCoV-HKU1) and 3 high pathogenic CoVs (SARS-CoV, MERS-CoV, and SARS-CoV-2), can infect humans^8–12^. To date, several strategies have been adopted for the development of anti-SARS-CoV-2 therapeutics by targeting viral spike (S) protein (S1 and S2 subunits), viral enzymes (PLpro, 3 CLpro, RdRp and helicase)^13^, and some structure proteins^13^. Generally, small molecular inhibitors with oral bioavailability are more suitable for intracellular targets, i.e., viral proteases, by the necessity of cell permeability. One inhibitor of main protease (M^pro^)/3C-like protease (3CLpro), Paxlovid™, was recently approved by the US FDA as an oral drug for treatment of SARS-CoV-2 infection^14^. However, instances of reinfection after completing the recommended course of Paxlovid are reported^15^, and recent studies show that this type of M^pro^ inhibitors tend to induce rapid drug resistance^16,17^.

SARS-CoV-2 neutralizing antibodies (nAbs) generally target RBD in S1 subunit^18–21^. However, nAbs lack oral bioavailability and lose neutralizing activity against SARS-CoV-2 variants that escape immune surveillance.

Jiang’s group previously identified a series of pan-CoV fusion inhibitors, such as EK1 peptide and EK1C4 lipopeptide, targeting the heptad repeat 1 (HR1) domain in S2 subunit of SARS-CoV-2 S protein with highly potent antiviral activity against all HCoVs tested^22,23^, demonstrating the potential of using S protein to develop pan-antiviral inhibitors. de Vries et al.^24^ synthesized a dimeric lipopeptide [SARS_HRC_-PEG_4_]_2_-chol, and with daily intranasal administration to SARS-CoV-2 ferrets, it could completely prevent SARS-CoV-2 direct-contact transmission with limited toxicity. Despite providing excellent inhibitory against SARS-CoV-2 virus and broad-spectrum antiviral activity^22,23,25^, however, these peptides generally suffer from low enzymatic stability and poor oral bioavailability. Therefore, this study aimed to identify peptide-based pan-CoV fusion inhibitors with high proteolytic enzyme stability and good oral bioavailability.

Cai’s group has established several cyclic γ-AApeptide-based one-bead-two-compound (OBTC) combinatorial libraries in which the cyclic γ-AApeptides possess high proteolytic enzyme stability and good oral availability^26–30^. Through screening these OBTC libraries, several important hits, such as cyclic γ-AApeptides targeting EphA2, EGFR and HER2 were identified^27,30,31^, suggesting that these libraries can be used for identification of γ-AApeptide-based pan-CoV fusion inhibitors with oral bioavailability.

Here, we (Jiang’s and Cai’s groups) worked together to screen a cyclic γ-AApeptide-based OBTC combinatorial library against SARS-CoV-2 S protein, and after the first screening, we identified **S-20**, a hit with potent fusion and entry inhibitory activity, but moderate selectivity index (SI). Through further modification of the hit, we found that the analog compound, **S-20-1**, exhibited potent fusion and entry inhibitory activity against SARS-CoV-2 and its variants as well as other HCoVs, such as HCoV-OC43, and had exceptionally high SI. **S-20-1** also demonstrated excellent in vivo efficacy by potently inhibiting both HCoV-OC43 and SARS-CoV-2 infections in mice and good in vivo safety profiles. Most importantly, **S-20-1** was highly resistant to proteolytic degradation and exhibited favorable oral bioavailability, suggesting a great potential to be further developed as a therapeutic and prophylactic drug for treatment and prevention of infection by SARS-CoV-2 and its variants as well as other HCoVs.

## Results

### Library design, synthesis, and screening

Inspired by the backbone of the chiral peptide nucleic acid (PNA), we recently developed a class of peptidomimetic γ-AApeptides which shows remarkable resistance to proteolytic degradation, robust helical folding propensity, and promising applications in biomedical sciences^32–34^. The chemodiversity and modular synthesis of γ-AApeptides make them ideal candidates to create combinatorial libraries bearing unnatural ligands^26–30^. To date, macrocyclic γ-AApeptides have been identified to bind nucleic acids and proteins with high affinity and specificity^26–30^. Here, we screen a library of γ-AApeptides against S protein of SARS-CoV-2.

We first constructed an OBTC combinatorial library comprised of thioether-bridge-mediated cyclic γ-AApeptides as reported previously (Supplementary Fig. S1)^27^. They contained a diverse and random set of hydrophobic and charged side chain units, resulting in a theoretical diversity of 320,000 compounds, with each compound being encoded by an 8-mer peptide (Supplementary Fig. S2a). The OBTC library was incubated with His-tag SARS-CoV-2 S protein, followed by incubation with Dylight 488 6×-His tag monoclonal antibody (Supplementary Fig. S2b). Putative positive beads were microscopically identified from the library pool (Supplementary Fig. S3), and the encoding peptides were analyzed by tandem MS/MS of MALDI. The chemical structures of 43 putative hits were determined unambiguously (Supplementary Fig. S4).

### S-20-1, a modified cyclic γ-AApeptide, exhibited high fusion inhibitory activity and low cytotoxicity

**Fig. 1:**
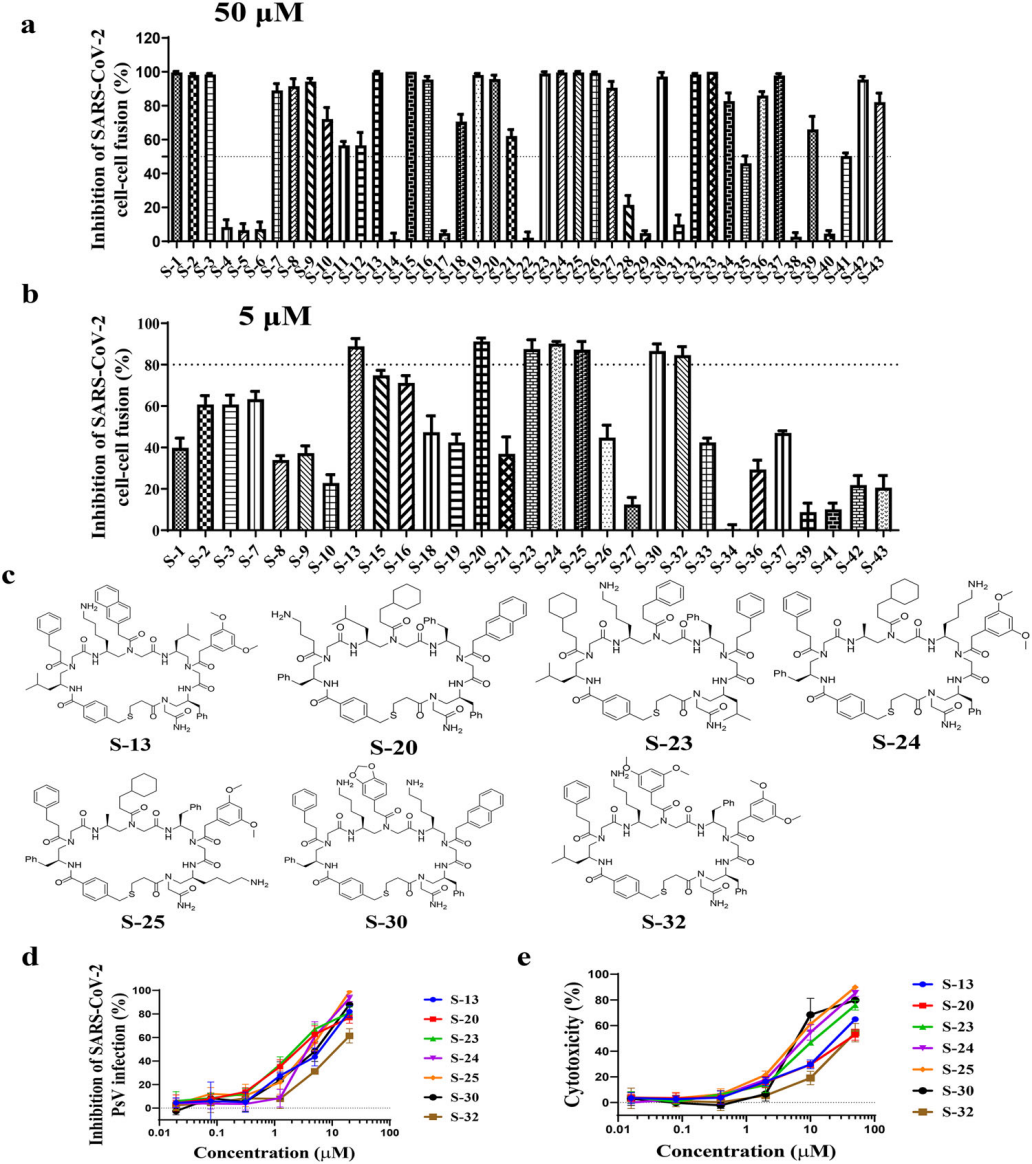
Screening for SARS-CoV-2 fusion and entry inhibitors from a cyclic γ-AApeptide library. Inhibition of cell–cell fusion mediated by the S protein of SARS-CoV-2 by putative hits at 50 μM (**a**) and 5 μM (**b**). The dot line in figures means the inhibition rate of 50% (**a**) and 80% (**b**). **c** Chemical structures of seven hits with inhibitory effect against SARS-CoV-2 S-mediated cell–cell fusion. **d** Inhibitory activity of hits (**S-13**, **S-20**, **S-23**, **S-24**, **S-25**, **S-30**, and **S-32**) from SARS-CoV-2 pseudovirus infection assay. **e** Cytotoxicity of hits (**S-13**, **S-20**, **S-23**, **S-24**, **S-25**, **S-30**, and **S-32**) on Huh-7 cells.

We first assessed the inhibitory activity of these 43 putative hits in vitro using a SARS-CoV-2 S protein-mediated cell–cell fusion assay established in our lab^22,23,25^. Under 50-μM concentration, 29 hits exhibited more than 50% inhibition (Fig. 1a). To confirm inhibitory activity and select final hits for further investigation, these compounds were tested again at a 5-μM concentration (Fig. 1b), revealing seven compounds (**S-13**, **S-20**, **S-23**, **S-24**, **S-25**, **S-30**, and **S-32**) still able to efficiently inhibit SARS-CoV-2 S-mediated cell–cell fusion (>80%). To further validate these compounds (Fig. 1c), we used our well-established SARS-CoV-2 pseudovirus (PsV) infection assay^22,23,25^ to assess their inhibitory activity of these compounds against SARS-CoV-2 PsV infection as indicated by the half maximal inhibitory concentration (IC_50_). Their cytotoxicity was simultaneously evaluated, and half-maximal cytotoxic concentration (CC_50_) was calculated to determine selectivity index (SI = CC_50_/IC_50_). All seven compounds showed excellent antiviral activities against SARS-CoV-2 PsV infection with IC_50_ of 1–5 µM (Fig. 1d; Supplementary Table S1). These compounds only exhibited cytotoxicity at noticeably higher concentration (Fig. 1e) and revealed decent selectivity with SI between 1.6 and 14.3 (Supplementary Table S1).

**Fig. 2:**
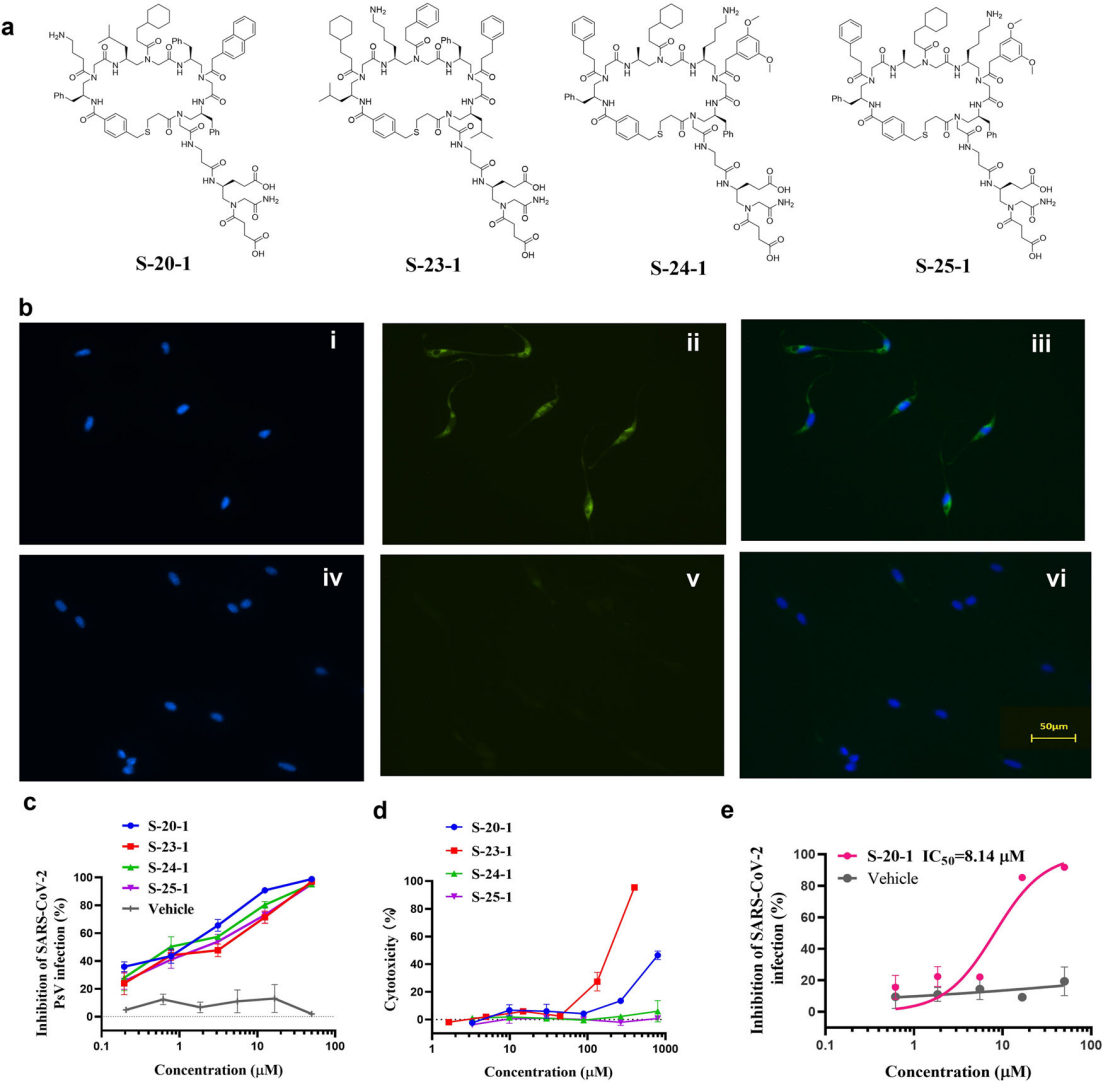
Identification of four modified cyclic γ-AApeptides with improved SARS-CoV-2 fusion/entry inhibitory activity and SI. **a** Chemical structures of four modified hits. **b** HeLa cells incubated with FITC labeled **S-20** (i–iii) and FITC labeled **S-20-1** (iv–vi) at 1 μM for 2 h, respectively, and then stained with DAPI. (i, iv) DAPI channel; (ii, v) FITC channel; (iii, vi) merged. **c** Inhibitory activity of 4 modified cyclic γ-AApeptides in PsV infection assays against SARS-CoV-2. **d** Cytotoxicity of 4 modified cyclic γ-AApeptides on Huh-7 cell line. **e** Inhibitory activity of **S-20-1** on authentic SARS-CoV-2 replication on Caco-2 cell line.

We next asked if SI of these hits could be further improved. We speculated that the low-to-moderate SI was caused by the ability of cyclic γ-AApeptides to cross the host cell membrane and potentially work on intracellular targets. Therefore, negative charges could be introduced to decrease cell permeability, thereby minimizing potential cytotoxicity^35,36^ and increasing SI. We then added two negative charges to each of four compounds **S-20**, **S-23**, **S-24**, **S-25** (Fig. 2a) that showed the best PsV inhibitory activity, and their ability to cross the cell membrane declined. No fluorescence was observed for **S-20-1** at 1 μM (Fig. 2b v) after incubation with HeLa cells compared to **S-20** (Fig. 2b ii) at the same condition, which showed strong fluorescence, demonstrating the abolishment of cell permeability of **S-20-1**.

The modified compounds were then tested for antiviral activity and cytotoxicity using the PsV assay (Fig. 2c) and cytotoxicity assay (Fig. 2d). As shown in Supplementary Table S2, modification of the compounds with negative charges did not significantly alter their antiviral activity. **S-20-1** even revealed a >3-fold better activity (IC_50_: 0.8 μM) compared with **S-20** (IC_50_: 2.9 μM), suggesting no effect of modification on the binding of these cyclic peptidomimetics toward S protein. Cytotoxicity of these compounds (Fig. 2d) was also largely diminished, leading to a remarkable improvement of SI (95 –>1000) (Supplementary Table S2). With IC_50_ of 0.8 μM and the CC_50_ of more than 800 μM (Supplementary Fig. S5a), **S-20-1** exhibited an exceptional SI (>1000). Based on its performance, we selected **S-20-1** and tested its inhibitory activity against authentic SARS-CoV-2 infection of Caco-2 cells. As anticipated, **S-20-1** effectively blocked authentic SARS-CoV-2 infection at the cellular level in a dose-dependent manner with an IC_50_ of 8.14 μM (Fig. 2e), consistent with the results from the PsV infection assay. **S-20-1** also exhibited low cytotoxicity on Caco-2 cells, with the CC_50_ of 692.7 μM (Supplementary Fig. S5b). Taken together, **S-20-1** was demonstrated to be a potent and highly selective inhibitor against SARS-CoV-2 infection.

### S-20-1 efficiently inhibited various SARS-CoV-2 variants in different cell lines

**Fig. 3:**
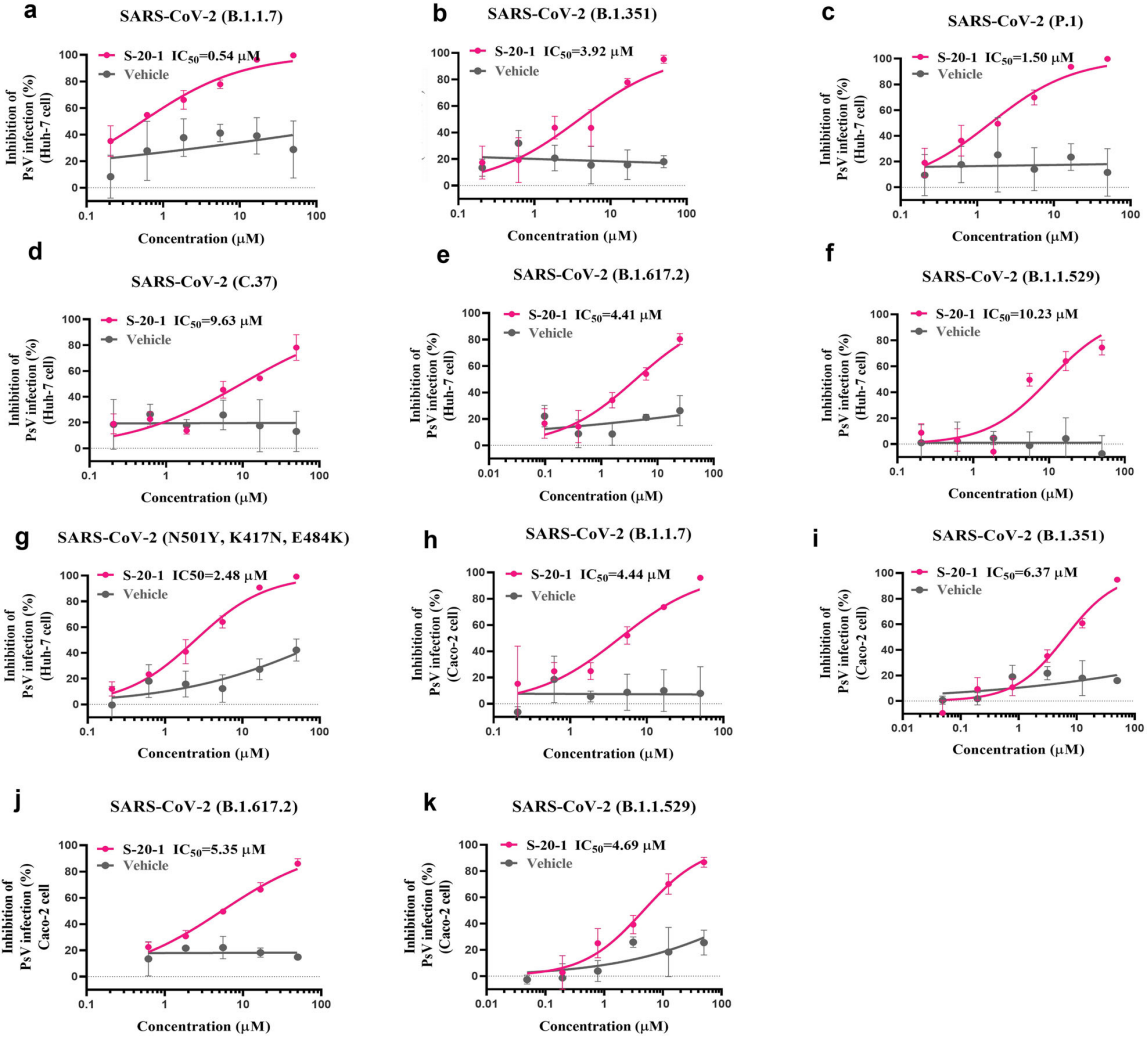
Inhibition of **S-20-1** against infection by pseudotyped SARS-CoV-2 variants in different cell lines. Inhibition of infection by PsV of SARS-variants on Huh-7 cells: **a** B.1.1.7 (Alpha), **b** B.1.351 (Beta), **c** P.1 (Gamma), **d** C.37 (Lambda), **e** B.1.617.2 (Delta), **f** B.1.1.529 (Omicron), **g** mutant with N501Y, K417N, and E484K mutation. Inhibition of infection by PsV of SARS-variants on Caco-2 cells: **h** B.1.1.7 (Alpha), **i** B.1.351 (Beta), **j** B.1.617.2 (Delta), and **k** B.1.1.529 (Omicron).

Next, we evaluated the in vitro efficacy of **S-20-1** against infection by SARS-CoV-2 variants, as cited previously, and in different cell lines. We found that **S-20-1** potently inhibited infection by pseudotyped B.1.1.7 (Fig. 3a), B.1.351 (Fig. 3b), P.1 (Fig. 3c), C.37 (Fig. 3d), B.1.617.2 (Fig. 3e), B.1.1.529 (Fig. 3f), and the mutant with N501Y, K417N, and E484K mutations (Fig. 3g) in the Huh-7 cell line with IC_50s_ values ranging from 0.54 to 10.23 μM. We also tested the anti-PsV activity of **S-20-1** against some of the most virulent SARS-CoV-2 variants in Caco-2 cells. Its inhibitory activity was consistent with that for Huh-7 cells, revealing IC_50s_ values ranging from 4.44 to 6.37 μM against B.1.1.7 (Fig. 3h), B.1.351 (Fig. 3i), B.1.617.2 (Fig. 3j) and B.1.1.529 (Fig. 3k). Together, **S-20-1** exhibited broad-spectrum inhibitory activity against predominant SARS-CoV-2 variants.

### S-20-1 potently inhibited cell–cell fusion-mediated by S proteins of 5 HCoVs and blocked infection by 4 pseudotyped HCoVs and 1 pseudotyped bat SARSr-CoV, as well as 2 authentic HCoVs

**Fig. 4:**
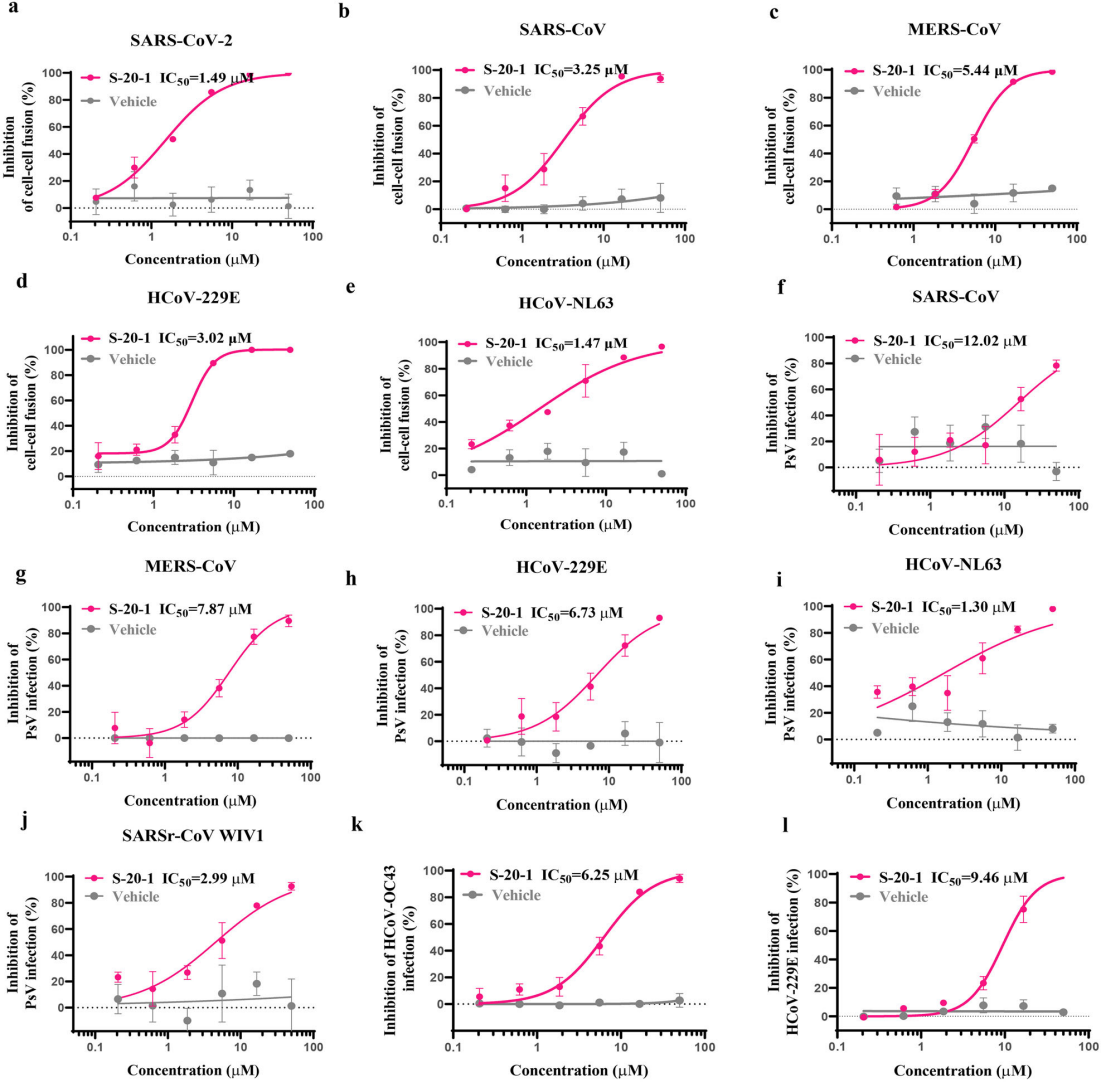
Inhibition of **S-20-1** against infection of divergent HCoVs and SARSr-CoV. Inhibitory activity of **S-20-1** on cell–cell fusion mediated by the S protein of SARS-CoV-2 (**a**) SARS-CoV (**b**) MERS-CoV (**c**) HCoV-229E (**d**) and HCoV-NL63 (**e**) Inhibitory activity of **S-20-1** against infection of pseudotyped SARS-CoV (**f**) MERS-CoV (**g**), HCoV-229E (**h**), HCoV-NL63 (**i**) and SARr-CoV W1V1 (**j**). Inhibitory activity of **S-20-1** against infection of authentic HCoV-OC43 (**k**) and HCoV-229E (**l**). RD cells and Huh-7 cells were infected with HCoV-OC43 (**k**) and HCoV-229E (**l**), respectively.

As an analog of **S-20**, **S-20-1** was expected to have strong binding affinity to S protein and have broad-spectrum antiviral activity against diverse HCoVs, including α-HCoV and β-HCoV, since these HCoV share conserved regions in S protein. First, we found that **S-20-1** potently inhibited cell–cell fusion mediated by S protein of SARS-CoV-2 (Fig. 4a), SARS-CoV (Fig. 4b), MERS-CoV (Fig. 4c), HCoV-229E (Fig. 4d) and HCoV-NL63 (Fig. 4e) with IC_50s_ ranging from 1.47 to 5.44 μM, confirming that **S-20-1** is a pan-HCoV fusion inhibitor. **S-20-1** also exhibited potent inhibitory activity against infection of pseudotyped SARS-CoV (Fig. 4f), MERS-CoV (Fig. 4g), HCoV-229E (Fig. 4h), HCoV-NL63 (Fig. 4i), and bat SARSr-CoV WIV1 (Fig. 4j) with IC_50s_ ranging from 1.30 to 12.02 μM, consistent with the result of SARS-CoV-2 PsV (Fig. 2c), indicating that **S-20-1** is a pan-CoV entry inhibitor. Finally, like authentic SARS-CoV-2 (Fig. 2e), authentic HCoV-OC43 and HCoV-229E infection in RD cells and Huh-7 cells was effectively inhibited by **S-20-1** with IC_50s_ of 6.25 μM (Fig. 4k) and 9.46 μM (Fig. 4l), respectively. The cytotoxicity of **S-20-1** on RD cells was also detected with the CC_50_ of 274.2 μM (Supplementary Fig. S5c). Overall, **S-20-1** demonstrates broad-spectrum antiviral activity against infection by HCoVs and SARSr-CoVs tested.

### Intranasally applied S-20-1 efficiently protected mice from infection by HCoV-OC43 and SARS-CoV-2 Delta variant

**Fig. 5:**
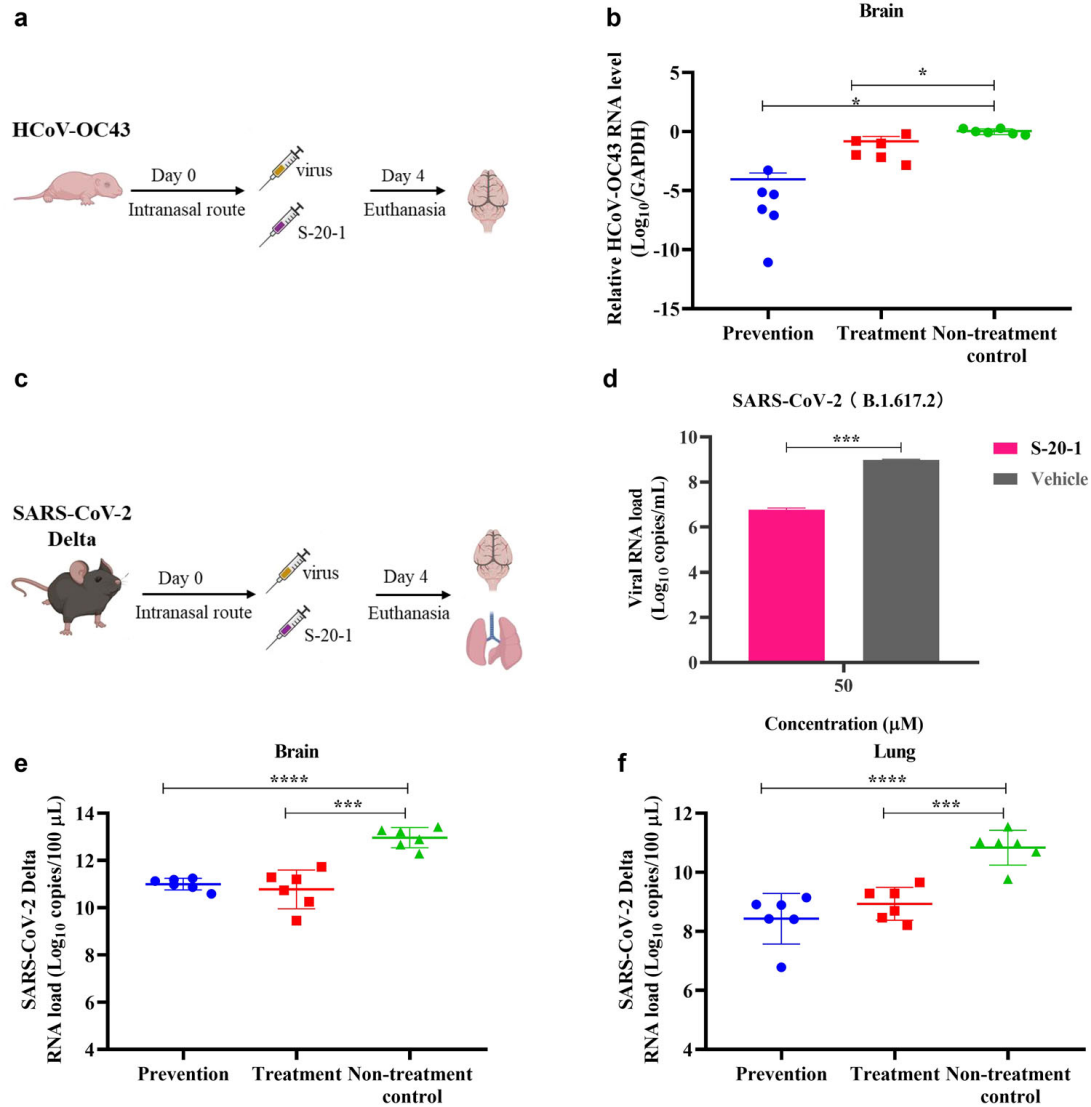
Prevention and treatment effect of **S-20-1** against mouse infection by HCoV-OC43 and SARS-CoV-2 Delta variant. **a** Schematic diagram of **S-20-1** administration and HCoV-OC43 challenge. **b** In vivo efficacy of **S-20-1** (80 mg/kg) against HCoV-OC43 infection in newborn mice. Viral RNA expression level in brain tissue of mice in each group on the 4th day post-infection was detected. **c** Schematic diagram of **S-20-1** administration and SARS-CoV-2 challenge. **d** Viral RNA expression level after incubation of **S-20-1** with authentic SARS-CoV-2 Delta on Caco-2 cells. **e**, **f** In vivo efficacy of **S-20-1** (60 mg/kg) against SARS-CoV-2 Delta variant infection in hACE2-transgenic C57BL/6 mice. Viral RNA expression level in mouse brain (**e**) and lung (**f**) of each group on the 4th day post-infection was detected. These data were analyzed by Student’s *t*-test (**d**) and One-way ANOVA (**b**, **e**, **f**).

To evaluate the protective effect of **S-20-1** in vivo, we first used an HCoV-OC43-infected mouse model to assess the prophylactic and therapeutic potential of **S-20-1** as an antiviral agent (Fig. 5a). **S-20-1** was administered to newborn mice in prevention or treatment group via the intranasal route at a single dose of 80 mg/kg 0.5 h pre- or post-challenge with HCoV-OC43 at 100 TCID_50_, respectively. At four days post-infection, mice were sacrificed, and brains were excised to evaluate viral load. As shown in Fig. 5b, relative HCoV-OC43 RNA level of both prevention and treatment group was significantly lower than that of non-treatment control group. Results showed that **S-20-1** could effectively protect newborn mice from infection of HCoV-OC43.

We then tested the protective efficacy of **S-20-1** on SARS-CoV-2 Delta variant-infected hACE2-transgenic mouse model, C57BL/6-Tgtn (CAG-human ACE2-IRES-LuciferaseWPRE-polyA)^37^ as described before (Fig. 5c). First, we assessed inhibitory activity against the SARS-CoV-2 Delta variant on Caco-2 cells in vitro, and viral load was significantly decreased about 2 logs (100-fold) at 50 µM concentration of **S-20-1** (Fig. 5d). Then we intranasally administered **S-20-1** at the dose of 60 mg/kg to hACE2-transgenic mice (female, eight weeks old) 0.5 h before (prevention group) or after (treatment group) at 10,000 pfu of SARS-CoV-2 Delta variant via the intranasal route. Viral load in the brain of mice in the prevention and treatment groups was 2.02 and 2.16 logs, respectively, lower than that in the non-treatment control group (Fig. 5e), while that in the lung of mice in the prevention and treatment groups was 2.5 logs and 2.3 logs, respectively, lower than that in the non-treatment control group (Fig. 5f). Therefore, intranasally administered **S-20-1** exhibited prophylactic and therapeutic effect against SARS-CoV-2 Delta infection.

### S-20-1 inhibited SARS-CoV-2 infection at the early stage of viral entry

**Fig. 6:**
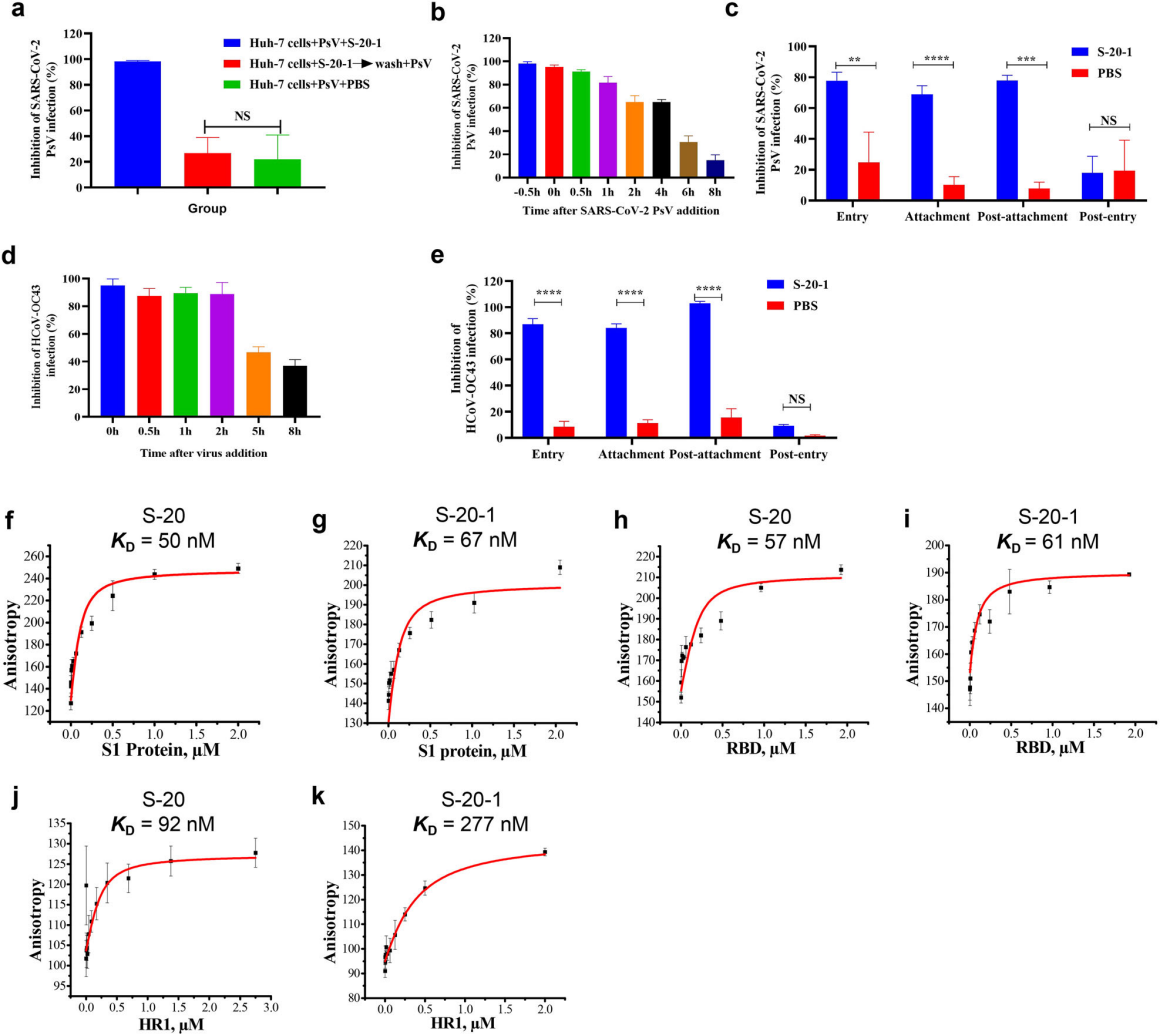
Inhibition of SARS-CoV-2 infection by **S-20-1** that specifically targets at RBD and HR1 domain at the early stage of viral entry. **a** In PsV infection assay, Huh-7 cells were pretreated with **S-20-1** at 50 µM at 37 ^o^C for 1 h, washed with PBS to remove unbound **S-20-1**, and infected with SARS-CoV-2 at 37 ^o^C. In time-of-addition assays, Huh-7 and RD cells were treated with **S-20-1** at the indicated time points before or after addition of pseudotyped SARS-CoV-2 (**b**) and authentic HCoV-OC43 (**d**), respectively. Supernatants containing free **S-20-1** and viral particles were removed 12 h later. A series of well-established assays were performed to confirm the stage at which **S-20-1** blocked entry of SARS-CoV-2 or HCoV-OC43 into target cells. Data were analyzed with One-way ANOVA (**a**) and Two-way ANOVA (**c**, **e**). NS means no significance. Affinity of binding between **S-20** and S1 (**f**), **S-20-1** and S1 (**g**), **S-20** and RBD (**h**), **S-20-1** and RBD (**i**), **S-20** and HR1 (**j**), or **S-20-1** and HR1 (**k**), was determined by fluorescence polarization.

To gain mechanistic insight of **S-20-1** against SARS-CoV-2 infection, we first used the time-of-removal assay to determine whether the inhibitory activity of **S-20-1** resulted from binding to virus or host cell surface to block SARS-CoV-2 entry. We incubated **S-20-1** with Huh-7 cells at 37 ^o^C for 1 h and then washed cells with PBS before SARS-CoV-2 was added. No inhibitory activity was observed after washing (Fig. 6a), suggesting that **S-20-1** targets virus, not host cells. Next, **S-20-1** was added to Huh-7 cells at different time points before, during, and after SARS-CoV-2 infection to determine affected stage of the viral life cycle. As shown in Fig. 6b, **S-20-1** exhibited more than 80% inhibition of SARS-CoV-2 infection when added 0.5 h before, at the same time (0 h), or 0.5 and 1 h after the addition of virus. The inhibitory activity was then gradually decreased to ~60% at 2 h and 4 h, 35% at 6 h, and 15% at 8 h, indicating that **S-20-1** may target the early stage of the virus life cycle. Next, we used a previously reported assay by adjusting the temperature to distinguish the process of entry, post-entry, attachment, and post-attachment^38^. As shown in Fig. 6c, **S-20-1** could inhibit 80%, 70%, and 75% in the entry stage, attachment stage, and post-attachment stage, respectively, with no effect at the post-entry stage, in good agreement with its targeting at the early fusion stage. We also assessed the inhibitory activity of **S-20-1** against HCoV-OC43 infection with the same assays described above. As shown in Fig. 6d, e, **S-20-1** exhibited results similar to those when SARS-CoV-2 was tested, suggesting that **S-20-1** targets the early entry stage of SARS-CoV-2, HCoV-OC43, and possibly other HCoVs.

The binding affinity of **S-20** and **S-20-1** toward various subunits in S protein was then determined by fluorescence polarization assays^33^. We obtained FITC-labeled cyclic γ-AApeptides **S-20** and **S-20-1** successfully (Supplementary Figs. S6, S7, and Table S3). Both compounds exhibited excellent binding affinity toward S1 subunit with *K*_D_ of 50 nM (Fig. 6f) and 67 nM (Fig. 6g), respectively. This similarity confirmed that modification of **S-20** with negative charges did not change its binding activity to S1 protein. We next measured the binding affinity of both compounds toward RBD, revealing *K*_D_ values of 57 nM (Fig. 6h) and 61 nM (Fig. 6i), respectively, suggesting that both **S-20** and **S-20-1** mainly target RBD on S1 subunit, which may account for their excellent inhibitory activity against SARS-CoV-2 in vitro. However, potent binding affinity to S1 subunit alone could not plausibly explain why **S-20-1** exhibited broad-spectrum antiviral activity against various HCoVs, as the S1 subunit is not well conserved in S protein. Recalling that Jiang’s group had previously identified EK1 peptide and EK1C4 lipopeptide targeting the HR1 domain in S2 subunit of SARS-CoV-2 S protein^22,23,25^, we speculated that **S-20-1** might also bind to this domain. Indeed, we found that **S-20** and **S-20-1** bound with HR1 domain tightly with *K*_D_ values of 92 nM (Fig. 6j) and 277 nM (Fig. 6k), respectively, possibly explaining the broad-spectrum activity of **S-20-1** toward various HCoVs. Interestingly, neither **S-20** nor **S-20-1** bound to the HR2 domain (Supplementary Fig. S8), which is consistent with results from pan-CoV fusion inhibitor EK1 peptide that binds with HR1, but not HR2. These results suggest that **S-20-1** inhibit SARS-CoV-2 infection, possibly by binding RBD in S1 subunit and HR1 region in S2 subunit of S protein on virus separately. Co-crystallographic analysis of the **S-20-1**/S protein complex should be performed in the future, in order to determine whether or not **S-20-1** can bind RBD and HR1 regions in one S protein simultaneously.

### Docking of S-20, which shares the active group of S-20-1, with RBD or HR1

Using the Schrödinger Glide docking program^30^, we could not dock **S-20-1** with either RBD or HR1 because of **S-20-1**’s long tail. When we reperformed the docking analysis with **S-20**, which has no tail, to mimic **S-20-1**, we found that **S-20** could bind with both RBD and HR1 via a number of hydrophilic and hydrophobic interactions.

**Fig. 7:**
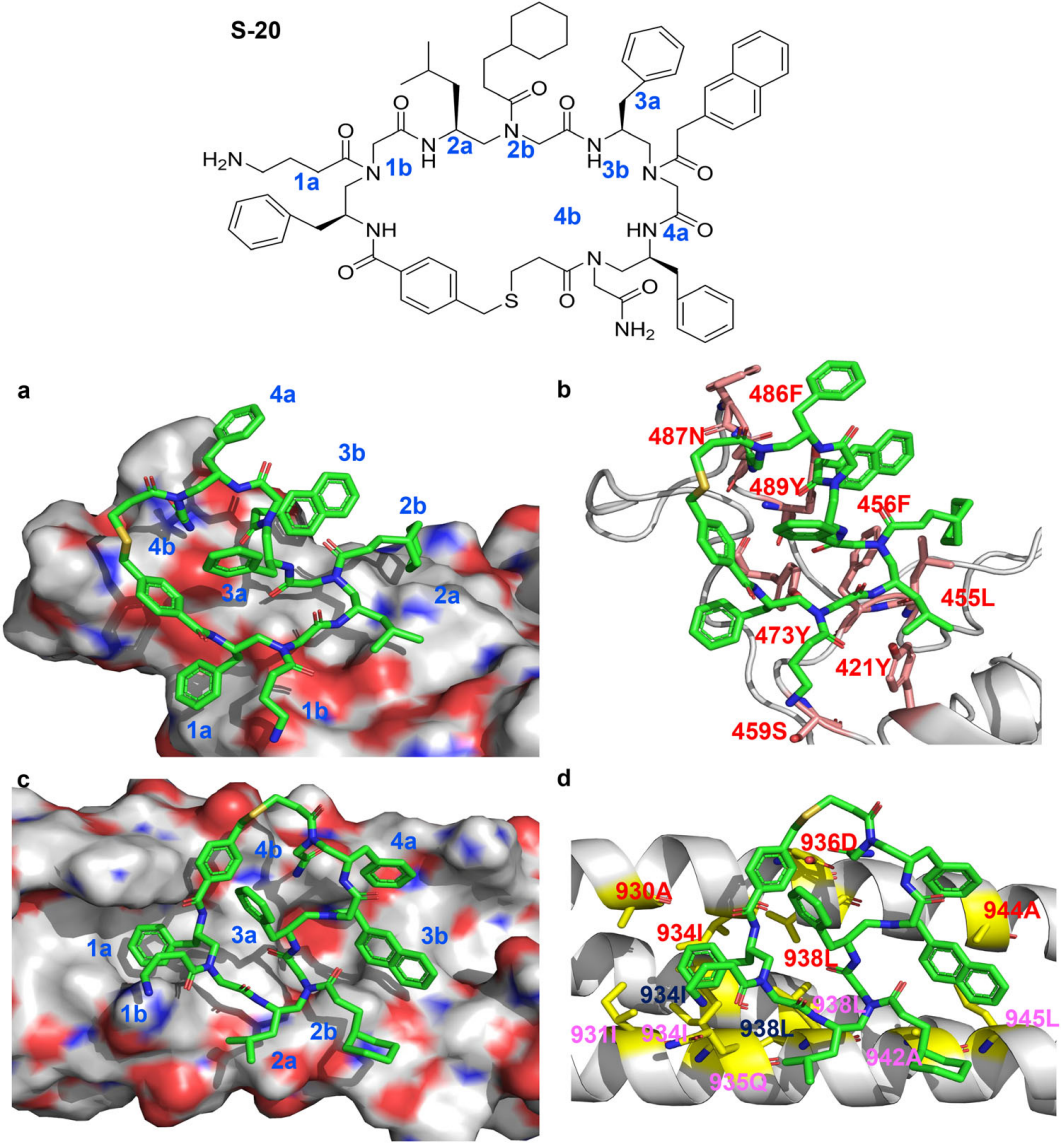
The chemical structure of S-20 and molecular docking analysis of the interaction between S-20 and its potential target sites. Side chains of **S-20** are designated by **a** (chiral side chain) or **b** (acyl side chain) in each AApeptide building block, respectively. Residues of HR1 from different helical chains are shown in red, black, and purple, respectively.

As shown in Fig. 7a, b, the hydrophobic side chains of 1a, 2a, 2b, 3b, and 4a of **S-20** could form either Pi-Pi interaction or hydrophobic interactions with either one or more residues of 473Y, 421Y, 455L, 456F, 489Y and 486F on RBD owing to close contacts. In addition, cationic 1b and hydrophilic 4b may form hydrogen bonding with 459S and 487N, respectively. On the other hand, the arrangement of the hydrophobic and hydrophilic groups on **S-20** also enables its favorable binding with the residues on HR1 (Fig. 7c, d). For instance, 1a of **S-20** deeply inserted into the hydrophobic domain formed by 930A, 931I, 934I, and 938L from three HR1 chains on the HR1/HR2 fusion core. Additionally, 2b, 3b, and 4a potentially adopted hydrophobic contacts with 942A, 945L, and 944A across different HR1 chains. Hydrophilic interactions between **S-20** and HR1 could also be identified, including potential hydrogen bonds forming between 1b and 935Q, 4b and 936D, respectively. Most residues in HR1 are highly conserved among HCoVs, including SARS-CoV-2, SARS-CoV, MERS-CoV, HCoV-229E, and HCoV-NL63/OC43, which may explain why **S-20** and **S-20-1** have broad-spectrum inhibitory activity against divergent HCoVs like EK1 peptide. Overall, the computational simulation is highly consistent with the experimental data, potentially providing guidance to optimize and develop more potent derivatives.

### S-20-1 was resistant to various proteolytic enzymes in blood

We next assessed the metabolic stability of **S-20-1** in the presence of proteinase K and trypsin. As shown in Supplementary Fig. S9a, b, the inhibition of SARS-CoV-2 PsV infection showed no decrease within 4-h incubation of **S-20-1** in the presence of proteinase K and trypsin. Next, **S-20-1** was incubated for 24 h with Pronase, a broad-specificity mixture of proteases extracted from Streptomyces griseus, followed by analysis with RP-HPLC. **S-20-1** was remarkably stable and showed no noticeable degradation, even at 24 h (Supplementary Fig. S9c, d), indicating its high resistance to various proteolytic enzymes in blood.

### S-20-1 possessed favorable passive permeability to the blood brain barrier (BBB) and gastrointestinal tract membranes, suggesting good oral bioavailability

**Fig. 8:**
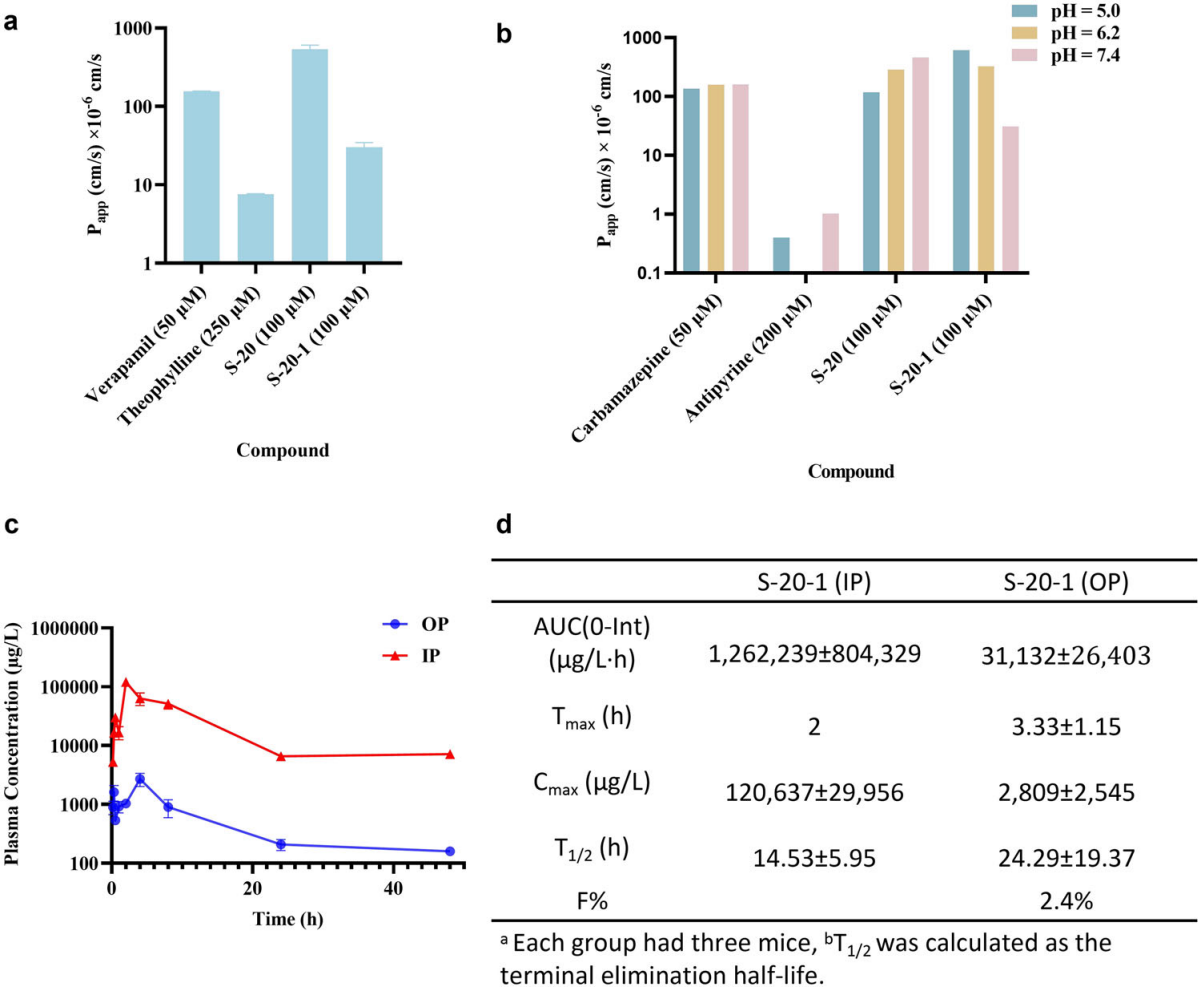
Evaluation of membrane passive permeability, oral bioavailability, and PK profiles of S-20-1 in mouse model. **a** PAMPA-BBB assay for standards, **S-20**, and **S-20-1** at pH 7.4. **b** PAMPA-GIT assay for standards, **S-20**, and **S-20-1** at different pH conditions. **c** Time-concentration plot of **S-20-1** in PK study. Plasma concentration and time curve following intraperitoneal (IP) (red) and oral administration (OP) (blue) administration of 50 mg/kg **S-20-1** in C57BL/6 mice (data indicated are means ± SD, *n* = 3). **d** Pharmacokinetics parameters of **S-20-1** over 48 h in mice.

The parallel artificial membrane permeability assay (PAMPA) is a high-throughput screening (HTS) technique to predict passive permeability by numerous different biological membranes, such as the gastrointestinal tract (GIT), blood brain barrier (BBB), and dermal layer^39^. Here, we employed PAMPA-BBB to evaluate the ability of **S-20-1** to penetrate the BBB and PAMPA-GIT to determine the gastrointestinal absorption rate and thus predict the oral bioavailability of **S-20-1**. For the PAMPA-BBB assay, we used Verapamil as positive control and Theophylline as negative control. The P_app_ values for favorable, medium, and low permeabilities are expected to be >20 × 10^−6^ cm/s, 1–20 × 10^−6^ cm/s and <1 × 10^−6^ cm/s, respectively. Surprisingly, **S-20** and **S-20-1** at 100 μM displayed favorable permeability with P_app_ values of 536 × 10^−6^ cm/s and 30 × 10^−6^ cm/s, respectively, while Verapamil (positive control) at 50 μM and Theophylline (negative control) at 250 μM exhibited P_app_ values of 155 × 10^−6^ and <10 × 10^−6^ cm/s, respectively (Fig. 8a). These results suggest that both **S-20** and **S-20-1** can effectively pass through BBB, which may explain why **S-20-1** showed strong protection against SARS-CoV-2 infection in mouse brain.

For the PAMPA-GIT assay, we used Carbamazepine and Antipyrine as the positive and negative controls, respectively, as Carbamazepine is fully orally bioavailable with favorable permeability at pH 5.0, 6.2, and 7.4 with P_app_ values of 135 × 10^−6^ cm/s, 158 × 10^−6^ cm/s and 160 × 10^−6^ cm/s, respectively, while Antipyrine is poorly orally bioavailable with low P_app_ values at different pH values. **S-20-1** displayed favorable permeability at pH 5.0, 6.2, and 7.4 with P_app_ at 616 × 10^−6^ cm/s, 326 × 10^−6^ cm/s and 31 × 10^−6^ cm/s, respectively (Fig. 8b). These results suggest that **S-20-1** may have a higher absorption rate under fed conditions than that in fasted conditions. Therefore, **S-20-1** is expected to have potential oral bioavailability.

### S-20-1 exhibited excellent pharmacokinetic (PK) profile and oral bioavailability tested in mouse model

To exploit the in vivo stability and oral bioavailability of **S-20-1**, we investigated its pharmacokinetics (Fig. 8c) by administering **S-20-1** in C57BL/6 mice via intraperitoneal (IP) and oral administration (OP) of **S-20-1** at 50 mg/kg over 48 h, respectively. For IP administration, **S-20-1** demonstrated excellent PK parameters with a long half-life (T_1/2_) of 14.53 h and a high peak concentration (C_max_) of 120,637 µg/L (Fig. 8d; Supplementary Fig. S10). For OP administration, **S-20-1** exhibited even longer half-life (T_1/2_ = 24.29 h) and an excellent oral bioavailability of ~2.4%, compared to IP.

### S-20-1 had good in vivo safety profiles in mouse model

**Fig. 9:**
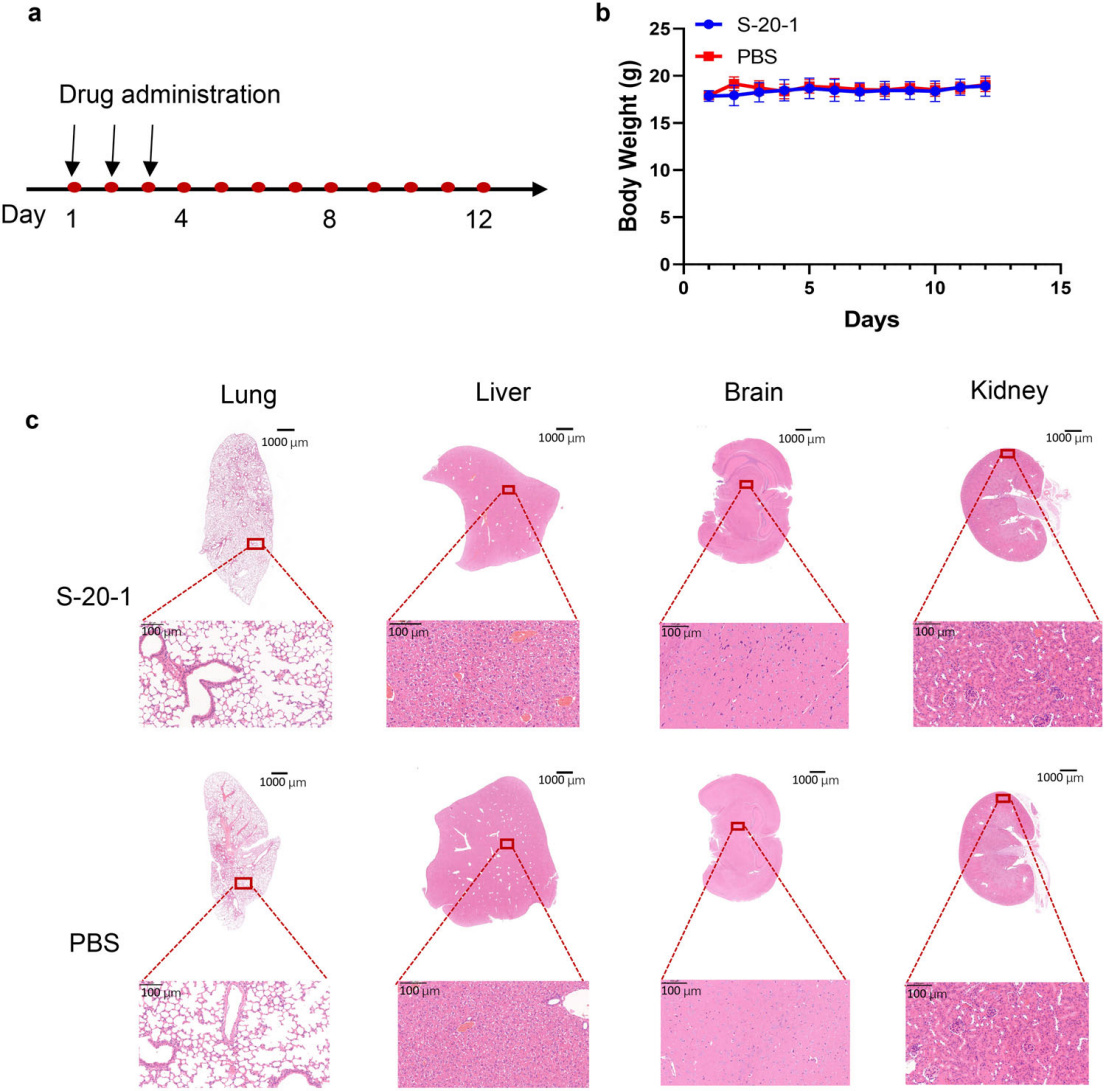
In vivo safety evaluation of **S-20-1**. **a** Flow diagram of in vivo safety experiments. **b** Body weight changes of mice administered with **S-20-1** or PBS intranasally. **c** Histological changes of mouse lung, liver, brain, and kidney after administration with **S-20-1** or PBS intranasally. Tissues were stained with H&E. The scale bar shown in slides were 1000 µm and 100 µm, respectively.

Eight-week-old Balb/c mice were used to test the in vivo safety of **S-20-1**. Mice were administered with **S-20-1** intranasally once daily for three days, and their body weight was monitored every day for 12 days (Fig. 9a). The body weight of mice in both **S-20-1** and PBS groups exhibited no significant changes (Fig. 9b). We then euthanized the mice on the 12^th^ day (Fig. 9a) and collected their liver, lung, kidney, and brain tissues. Histological sections of the tissues were stained with hematoxylin and eosin (H&E) and examined microscopically. Both **S-20-1** and PBS groups showed similar histological features (Fig. 9c). No inflammatory changes were observed in these tissues, suggesting that **S-20-1** is safe.

## Discussion

SARS-CoV-2 S protein consists of several important targets for the development of viral fusion and entry inhibitors^32^. For example, nAbs and other proteins inhibit SARS-CoV-2 infection by binding RBD in S1 subunit and blocking viral attachment to the receptor on the host cell^18–21^. Peptides derived from the HR2 domain, such as 2019-nCoV-HR2P, suppress SARS-CoV-2 fusion and entry by interacting with the HR1 in S2 subunit and interfering with the interaction between HR1 and HR2 to form the fusion-active 6-HB^40^.

Jiang’s group previously identified a series of pan-CoV fusion inhibitors, such as EK1 peptide and EK1C4 lipopeptide, targeting the HR1 domain in S2 subunit of SARS-CoV-2 S protein with highly potent antiviral activity against all HCoVs tested^22,23^. Therefore, these peptide-based pan-CoV fusion inhibitors can be developed for intranasally applied therapeutics for treatment of SARS-CoV-2 infection^37^. However, their future clinical use may not be preferably selected because of their lack of oral bioavailability. Meanwhile, Cai’s group previously established several cyclic γ-AApeptide-based OBTC combinatorial libraries in which the cyclic γ-AApeptides possess high proteolytic enzyme stability and potent biological activity^26–30^. For example, several cyclic γ-AApeptides were identified to target EphA2, EGFR, and HER2 with excellent binding affinity and specificity^27,30,31^. Therefore, it is feasible to identify some γ-AApeptide-based pan-CoV fusion inhibitors with oral bioavailability.

Here, Jiang’s and Cai’s groups worked together to identify cyclic γ-AApeptide-based pan-CoV fusion and entry inhibitors with oral bioavailability. More specifically, a cyclic γ-AApeptide-based OBTC combinatorial library was first screened against SARS-CoV-2 S protein and 43 active beads with SARS-CoV-2 S protein-mediated cell–cell fusion inhibitory activity at 50 μM were identified. Upon validation, seven potential hits were selected for further evaluation using SARS-CoV-2 PsV infection assay. The four best hits with better PsV inhibitory activity, including **S-20**, were selected for modification. We found that one of the derivative compounds, **S-20-1**, exhibited the most potent inhibitory activities against infection by pseudotyped and authentic SARS-CoV-2 and highest SI (>1000).

Most importantly, **S-20-1** is highly resistant to proteolytic degradation (showing no noticeable degradation up to 24 h when it was incubated with Pronase) and has a long half-life (~24 h) with oral administration, which is much longer than that (~2 h) of nirmatrelvir through oral administration^41^. We believe that the following two reasons may explain why **S-20-1** with a small size has a long half-life: (1) the unnatural backbones in γ-AApeptides are highly resistant to enzymatic hydrolysis, and (2) the cyclization of γ-AApeptides can rigidify functional groups to further increase stability towards proteolysis. In addition, **S-20-1** possesses favorable oral bioavailability with P_app_ values of 30 × 10^−6^ cm/s. To further confirm its proteolytic stability, long half-life, and oral bioavailability, we will perform experiments to evaluate the prophylactic and therapeutic effects through the oral route once daily in the future. If these are confirmed, **S-20-1** has the potential to be used in combination with other orally applicable COVID-19 drugs with different mechanisms of action or targeting different proteins, such as M^pro^ inhibitors (e.g., Paxlovid)^42^. These combinations may have synergistic effect and raise the genetic barrier to drug resistance.

**Fig. 10:**
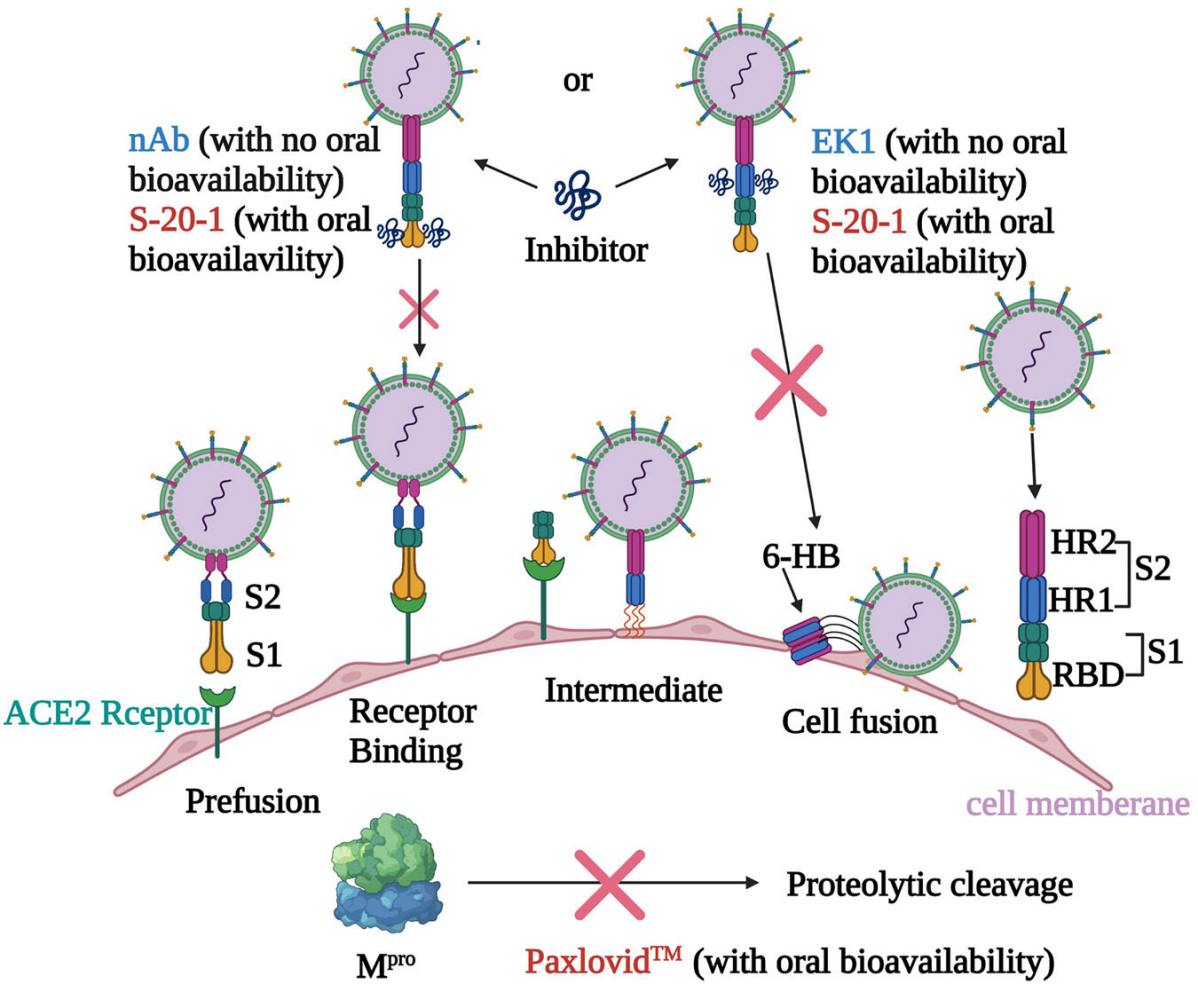
Proposed mechanism of action of **S-20-1** against SARS-CoV-2 infection. The entry of SARS-CoV-2 into the host cell is initiated by binding of RBD in S1 subunit of S protein to ACE2 (the receptor of SARS-CoV-2), which triggers the conformation change of S2 subunit of S protein and exposes the fusion intermediate structure consisting of HR1, HR2, and fusion peptide (FP). Then, HR1 and HR2 interact with each other to form 6-HB, bringing the viral and host cell membranes together for fusion. Like SARS-CoV-2 nAb and EK1 peptide, **S-20-1** is able to bind with RBD in S1 subunit and HR1 in S2 subunit to block viral attachment and fusion, respectively. Different from nAb and EK1 peptide, **S-20-1** also has oral bioavailability like Paxlovid™, noted above, which targets the intracellular main protease (M^pro^). However, **S-20-1** is superior to peptide- and lipopeptide-based pan-CoV fusion inhibitors because it is much more resistant to proteolytic enzymes and has a longer half-life than EK1, as well as good oral bioavailability. Therefore, **S-20-1** has better potential to be developed as an orally usable drug for treatment of SARS-CoV infection. The figure was created with BioRender.com.

Mechanistic studies suggested that **S-20-1** acts at the early entry stage of the viral life cycle, including attachment, post-attachment stages, and fusion stage, but not the post-entry stage. Further investigation demonstrates that **S-20-1** has dual targets in S protein, including RBD in the S1 subunit and HR1 in S2 subunit, suggesting that it inhibited SARS-CoV-2 fusion with and entry into the host cell by binding with RBD to block its interaction with the ACE2 receptor on the host cell, just like neutralizing antibodies, and interacting with HR1 to interfere with fusion activity and 6-HB formation, just like EK1 (Fig. 10). Of course, it is impossible to allow one cyclic peptide bind both RBD and HR1 at the same time because of its limit size. We believe that different **S-20-1** molecules may bind RBD and HR1 simultaneously or separately to inhibit viral infection. HR1 is a highly conserved domain in S protein of HCoVs, providing the basis of broad-spectrum anti-HCoV activity of **S-20-1** like the peptide-based pan-CoV fusion inhibitors EK1 and EK1C4^22,23^. Moreover, as **S-20-1** could potently bind with RBD and HR1 in the spike protein, it is expected to be hard to generate drug resistance in the clinical application.

In conclusion, based on our previous experience in developing peptide-based pan-CoV fusion inhibitors and cyclic γ-AApeptide-based protein binders, we herein identified a modified cyclic γ-AApeptide-based pan-CoV fusion and entry inhibitor, **S-20-1**. By targeting the RBD in S1 subunit and HR1 in S2 subunit of S protein, **S-20-1** exhibited potent and broad-spectrum inhibitory activity against infection by SARS-CoV-2, its variants, and other HCoVs, as well as bat SARSr-CoVs. It protected mice from infection of SARS-CoV-2 and HCoV-OC43 infection with a good in vivo safety profile. Most importantly, **S-20-1** was highly resistant to proteolytic degradation, and it exhibited long half-life and favorable oral bioavailability. These results suggested that **S-20-1** is a promising orally deliverable antiviral therapeutic and prophylactic candidate against current SARS-CoV-2 and its variants, as well as future emerging and re-emerging HCoVs.

## Materials and methods

### Materials

All chemicals were purchased from commercial suppliers and directly used without further purification. Fmoc-protected amino acids were purchased from Chem-impex and used for the building block preparation. TentaGel resin (0.23 mmol/g) used for OBTC library preparation was purchased from RAPP Polymere. Rink Amide-MBHA resin (0.55 mmol/g) used for the synthesis of cyclic γ-AA peptides was purchased from GL Biochem. Analysis and purification of cyclic γ-AA peptides was performed on the Waters Breeze 2 HPLC system and lyophilized on a Labcono lyophilizer. Purity of the compounds was determined to be >95% by analytical HPLC. The mass of each compound was confirmed by high-resolution mass spectrometry detected by Agilent 6220 using electrospray ionization time-of-flight (ESI-TOF). MS/MS analysis for the decoding sequence was obtained with an Applied Biosystems 4700 Proteomics Analyzer.

293T, RD, and Caco-2 cells were purchased from ATCC and stocked in our laboratory. Huh-7 cells were obtained from the Chinese Academy of Science Cell Bank (Shanghai, China). Caco-2 cells were cultured in MEM containing 10% FBS. Other cells were cultured with DMEM containing 10% FBS. HCoV-OC43 (VR-1558) and HCoV-229E (VR-740) were obtained from ATCC and propagated in our laboratory. SARS-CoV-2 (nCoV-SH01, GenBank number: MT121215.1) and SARS-CoV-2 Delta variant were isolated by Fudan University.

### OBTC library synthesis, screening, and analysis

The OBTC library was synthesized following our previous report^26–30^ and the scheme for library synthesis is provided in the Supplementary Information.

### Inhibition of pseudovirus infection

Assays for measuring the inhibitory activity of the compounds against pseudotyped coronavirus infection were conducted as previously described^43,44^. Plasmids encoding S protein of coronavirus, including SARS-CoV-2, SARS-CoV-2 variants (Alpha, Beta, gamma, lambda, Delta, Omicron), SARS-CoV, MERS-CoV, HCoV-OC43, HCoV-229E, SARSr-CoV WIV1, luciferase reporter vector (pNL4-3. Luc.R-E-), and plasmids encoding EGFP were maintained in our laboratory. For the package of pseudoviruses, pcDNA3.1- SARS-CoV-2-S and pNL4-3.Luc.R-E- were co-transfected into 293 T cells using Vigofect transfection reagent, and then the supernatants were changed with fresh medium containing 10% FBS. After 48 h, the supernatants containing pseudoviruses were collected, filtered with a 0.45 µm filter, and stocked. To determine the inhibitory activity of a given compound, target cells (Huh-7 cells) were seeded at 8000 per well in a 96-well plate and cultured at 37 °C for 12 h. The compound was diluted with DMEM without FBS, and then the same volume of pseudoviruses was added. Afterwards, the mixture was transferred into Huh-7 cells and incubated for 30 min. After 12 h, the mixture was replaced with fresh medium. Forty-eight h later, the cells were lysed with cell lysis buffer, and luciferase activity was detected with the Luciferase Assay System (Promega, Madison, WI, USA).

### Inhibition of authentic coronavirus infection

The inhibitory activity of **S-20-1** against authentic viruses was tested according to previous study^45^. In brief, **S-20-1** was serially diluted with DMEM without FBS. Then 100 TCID50 of virus were mixed with diluted **S-20-1**. After incubation for 30 min, the mixtures were transferred to target cells (RD for HCoV-OC43, Huh-7 for HCoV-229E, and Caco-2 for SARS-CoV-2 and SARS-CoV-2 Delta). The medium was changed 12 h later, and cell viability was detected with CCK8 kit (HCoV-OC43 and HCoV-229E). For SARS-CoV-2 and SARS-CoV-2 Delta, the supernatants were collected after 48 h. The viral RNA load was tested as previously reported^45^. Briefly, the viral RNA was extracted with RNA extraction kit (Transgene, China). Then the N gene of SARS-CoV-2 was tested by real-time RT-PCR. The sequence of primer and probe follows:

Forward: GGGGAACTTCTCCTGCTAGAAT;

Reverse: CAGACATTTTGCTCTCAAGCTG

Probe: 5′-FAM-TTGCTGCTGCTTGACAGATT-TAMRA-3′

### Inhibition of S protein-mediated cell–cell fusion

The cell-cell fusion assay was established and performed as in previous study^46^. In brief, PAAV-IRES-EGFP S was transfected to 293 T cells to obtain effector cells expressing S protein of coronaviruses, including SARS-CoV-2, SARS-CoV, MERS-CoV, HCoV-229E, and HCoV-NL63, and GFP. Then serially diluted **S-20-1** was mixed with effector cells, and the mixture was transferred to Huh-7 cells (target cells). For SARS-CoV and NL63 S-mediated cell–cell fusion assay, trypsin (80 mg/mL) was added to the mixture. After incubation for 2–4 h, fused cells were counted, and the fusion rate was calculated to determine inhibitory activity.

### Time-of-addition assay and time-of-removal assay

As in previous study^38,47^, for time-of-addition assay, Huh-7 cells (for pseudotyped SARS-CoV-2) and RD cells (for HCoV-OC43) were seeded into a 96-well plate at 10,000 per well, respectively. **S-20-1** was added at the final concentration of 50 µM 0.5 h before or 0, 0.5, 1, 2, 4, 6, and 8 h after addition of SARS-CoV-2 pseudoviruses or HCoV-OC43 (100 TCID_50_). The inhibitory activity of **S-20-1** was determined as described above.

For time-of-removal assay, **S-20-1** was added to Huh-7 cells to incubate at 37 °C for 1 h. After **S-20-1** was removed, SARS-CoV-2 pseudovirus was added to infect cells. **S-20-1** was not removed from the group set as control. The medium was changed 12 h later, and luciferase activity was tested as described above.

### Assays for detecting viral entry, attachment, post-attachment, and post-entry

Viral entry assay was performed as previously described^38,47^, Briefly, **S-20-1** and virus were added to target cells at 37 °C for 1 h, and then cells were washed with cold PBS three times. To perform the viral attachment assay, the mixture of **S-20-1** and virus was added to target cells to incubate for 1 h at 4 °C before washed with cold PBS. For the post-attachment assay, virus was incubated with target cells at 4 °C for 1 h. Then the cells were thoroughly washed with cold PBS to remove unattached virus. **S-20-1** was added and incubated at 37 °C for an additional 1 h. The post-entry assay was performed like the post-attachment assay, except that virus was incubated with cells at 37 °C. The inhibition effects of **S-20-1** were detected as above.

### Cytotoxicity assay

Cytotoxicity of compound to cells (Huh-7 cells and Caco-2 cells) was tested as previously described^22^. Briefly, serially diluted compounds were added to target cells. After culture at 37 °C for 12 h, the medium was changed with fresh medium. Forty-eight h later, the supernatant was removed, and cell viability was analyzed with Cell Counting Kit (CCK-8). In a 96-well plate, 100 µL of diluted CCK-8 reagent were added to each well, and the absorbance was measured at 450 nm.

### Mouse pharmacokinetic studies

In two separate experiments, **S-20-1** was administered either OP or IP to C57BL/6 mice at the dose of 50 mg/kg, volume 150 µL. Following administration, 100 µL blood samples were collected at 10 min, 20 min, 30 min, 1 h, 2 h, 4 h, 8 h, 24 h and 48 h (*n* = 3 per time point, and each mouse was used for three time points; thus 9 mice were used for either OP or IP, making a total of 18 mice). After drug administration, 100 µL of blood were collected into 1.5-mL Eppendorf tubes containing 30 µL disodium EDTA (0.5 M, pH 8.0) and kept on ice until plasma collection (<30 min), followed by centrifugation at 4000 rpm/min for 10 min at 4 ^o^C. The supernatants were collected and stored at –80 ^o^C for future analysis. Serum samples of 50 µL were added to 135 µL acetonitrile and 15 µL glacial acetic acid. Samples were allowed to rest on ice for 15 min and then centrifuged at 10,000 rpm and 4 ^o^C for 15 min. Clarified supernatants were transferred to vials and analyzed by LC/MS/MS. PK parameters were obtained using PKSolver.

### Evaluation of the in vivo protective activity of S-20-1

The protective effect of **S-20-1** against coronavirus in vivo was performed according to previous study^22^. Animal studies were approved by the Institutional Laboratory Animal Care and Use Committee at Fudan University (Approval number: 20200821-002). For HCoV-OC43, infected newborn mice were established as previously described. Pregnant Balb/c mice (18 days) were separated into three groups after delivery of their offspring. Each group contained seven newborn mice. For mice in the prophylactic and therapeutic groups, **S-20-1** was administered through the intranasal route at 80 mg/kg before or after challenge with HCoV-OC43. At the 4th day post-infection, the newborn mice were dissected. The relative viral RNA expression level in brain was tested through RT-PCR and calculated as 2^(−ΔΔCt)^. The HCoV-OC43 RNA level was adjusted with mouse housekeeping gene GAPDH. The primer of HCoV-OC43 and GAPDHA follows:

OC43-S-Forward: GACACCGGTCCTCCTCCTAT;

OC43-S-Reverse: ACACTTCCCTTCAGTGCCAT;

GDPAH-Forward: TGCTGTCCCTGTATGCCTCTG;

GDPAH-Reverse: TTGATGTCACGCACGATTTCC.

For SARS-CoV-2 Delta, we used C57BL/6-Tgtn (CAG-human ACE2-IRES-LuciferaseWPRE-polyA)-transgenic mice infected with SARS-CoV-2^37^. Eight-week-old female hACE2 transgenic mice were challenged with SARS-CoV-2 Delta variant at 10,000 pfu via the intranasal route. For prevention and therapy groups, **S-20-1** was administered at the dose of 60 mg/kg through the intranasal route 30 min before or after viral challenge. Then the mice were euthanized at 4 days post-infection, and brains, lungs, and intestines were dissected. Viral RNA was extracted with TRIzol reagent according to the manual. Real-time RT-PCR was conducted to evaluate viral RNA load in tissues as described previously.

### Evaluation of in vitro proteolytic enzyme stability and in vivo safety of S-20-1

For stability, the resistance of **S-20-1** to proteinase K and trypsin was performed as previously described^44^. **S-20-1** was incubated with proteinase K (1 microunit/mL) for different time and then centrifuged at 500× *g* for 5 min to remove the proteinase K. To determine the stability of **S-20-1** against trypsin, **S-20-1** was incubated with trypsin (25 mg/mL) for different time, followed by addition of FBS to final proportion of 20% and heated at 56 °C for 30 min to inactivate trypsin. The inhibitory activity of treated **S-20-1** was tested on Huh-7 cells.

Eight-week-old mice (two groups, *n* = 6) were used to evaluate the safety of **S-20-1** in vivo. According to previous study^48^, 100 mg/kg **S-20-1** were intranasally administered to mice daily for three consecutive days. Then body weight was monitored for 12 days, followed by observing the behavior of mice. At 12^th^ day, mice were euthanized to harvest the brains, lungs, livers, and kidneys for H&E staining.

### Statistical analysis

Student’s *t*-test and Analysis of Variance (ANOVA) were used to compare the difference by GraphPad Prism in this manuscript. *P < 0.05, **P < 0.01, ***P < 0.001, and ****P < 0.0001.

## Supplementary information

Supplementary Information
