# Supplementary material for: A novel cyclic γ-AApeptide-based long-acting pan-coronavirus fusion inhibitor with potential oral bioavailability by targeting two sites in spike protein
Source: Cell Discov. 2022 Sep 8;8:88. doi: 10.1038/s41421-022-00455-6 (PMC9453727; doi:10.1038/s41421-022-00455-6)
Supplement: Supplementary file 1 — Supplementary Information [file 41421_2022_455_MOESM1_ESM.pdf]

A novel cyclic γ-AApeptide-based long-acting pan-coronavirus fusion inhibitor with potential oral bioavailability by targeting two sites in spike protein

Songyi Xue,[1,+] Xinling Wang,[2,+] Lei Wang,[1,] Wei Xu,[2] Shuai Xia,[2] Lujia Sun,[2] Shaohui Wang,[3] Ning Shen,[1] Ziqi Yang,[1] Bo Huang,[1] Sihao Li,[1] Chuanhai Cao,[1,4] Laurent Calcul,[1] Xingmin Sun,[3] Lu Lu,[2,*] Jianfeng Cai[1,*], and Shibo Jiang[2,*]

[1]Department of Chemistry, University of South Florida, 4202 E. Fowler Ave., Tampa, FL, United States.

[2]Key Laboratory of Medical Molecular Virology (MOE/NHC/CAMS), School of Basic Medical Sciences, Shanghai Frontiers Science Center of Pathogenic Microbes and Infection, Shanghai Institute of Infectious Disease and Biosecurity, Fudan University, Shanghai, China.

[3]Department of Molecular Medicine, Morsani College of Medicine, University of South Florida, Tampa, FL, United States.

[4]Department of Pharmaceutical Science, Taneja College of Pharmacy, University of South Florida, Tampa, FL 33612, USA.

+ These authors contributed to the work equally

*Correspondence: shibojiang@fudan.edu.cn, jianfengcai@usf.edu, and lul@fudan.edu.cn

Table of contents

Supplementary Fig. S1. 3
Supplementary Fig. S2. 4
Supplementary Fig. S3. 5
Supplementary Fig. S4. 9
Supplementary Fig. S5. 10
Supplementary Fig. S6. 11
Supplementary Fig. S7. 12
Supplementary Fig. S8. 13
Supplementary Fig. S9. 14
Supplementary Fig. S10. 15
Supplementary Table S1. 16
Supplementary Table S2. 16
Supplementary Table S3. 16
MATERIALS AND METHODS 17
1. Screening of One-Bead-Two-Compound library 17
2. Cell permeability assay 19
3. Fluorescence polarization assay 19
4. Molecular docking studies 20
5. PAMPA-BBB assay 20
6. PAMPA-GIT assay 21
7. Assessment of enzymatic stability of S-20-1 21
References 22

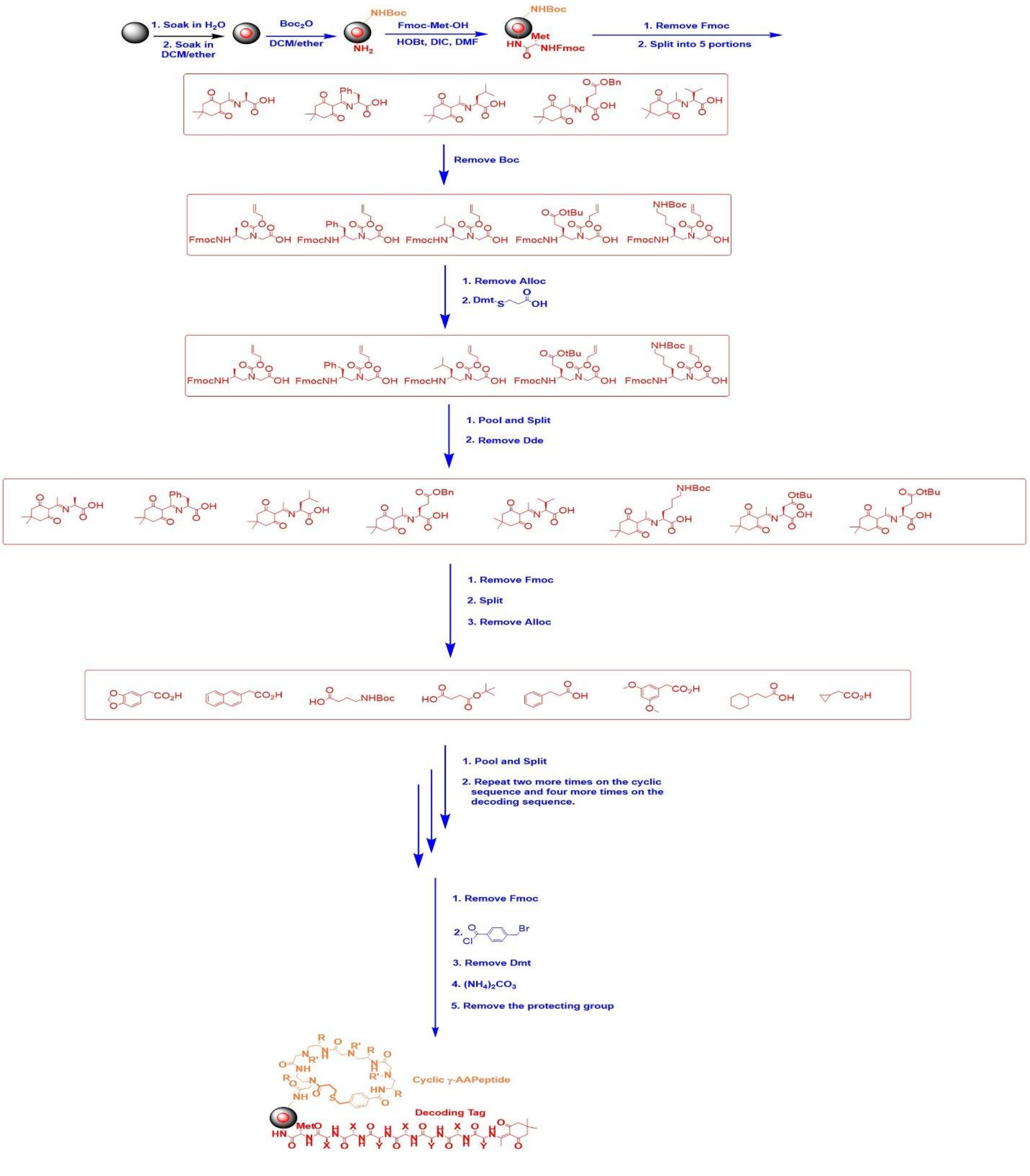

Supplementary Fig. S1. Preparation of the cyclic γ-AApeptides library.

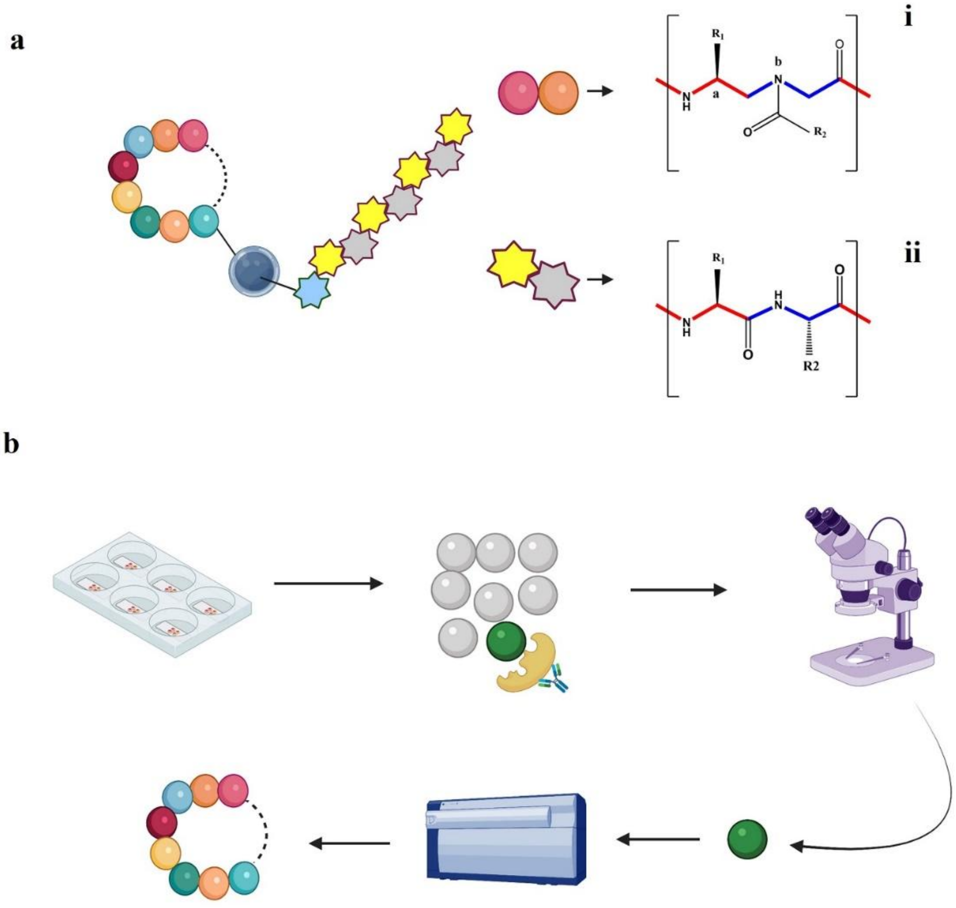

Supplementary Fig. S2. Library developed for inhibitors targeting SARS-CoV-2 S protein. (a) Schematic presentation of OBTC TentalGel beads. Chemical structures of γ-AApeptide (i) and α-peptide (ii). (b) Scheme showing the overall strategy. Briefly, the beads were incubated with blocking buffer (1% BSA in Tris buffer with 1000 × excess of E. coil lysate), His-tag SARS-CoV-2 S protein, and Dylight 488 6x-His Tag Monoclonal Antibody, respectively. Then the beads were transferred into 6-well plate. Following that, beads were observed under a fluorescence microscope and the beads emitting green fluorescence were picked up. After thorough wash, the decoding sequence were cleavage from the beads and analyzed by MALDI to determine the cyclic compounds’ structures.

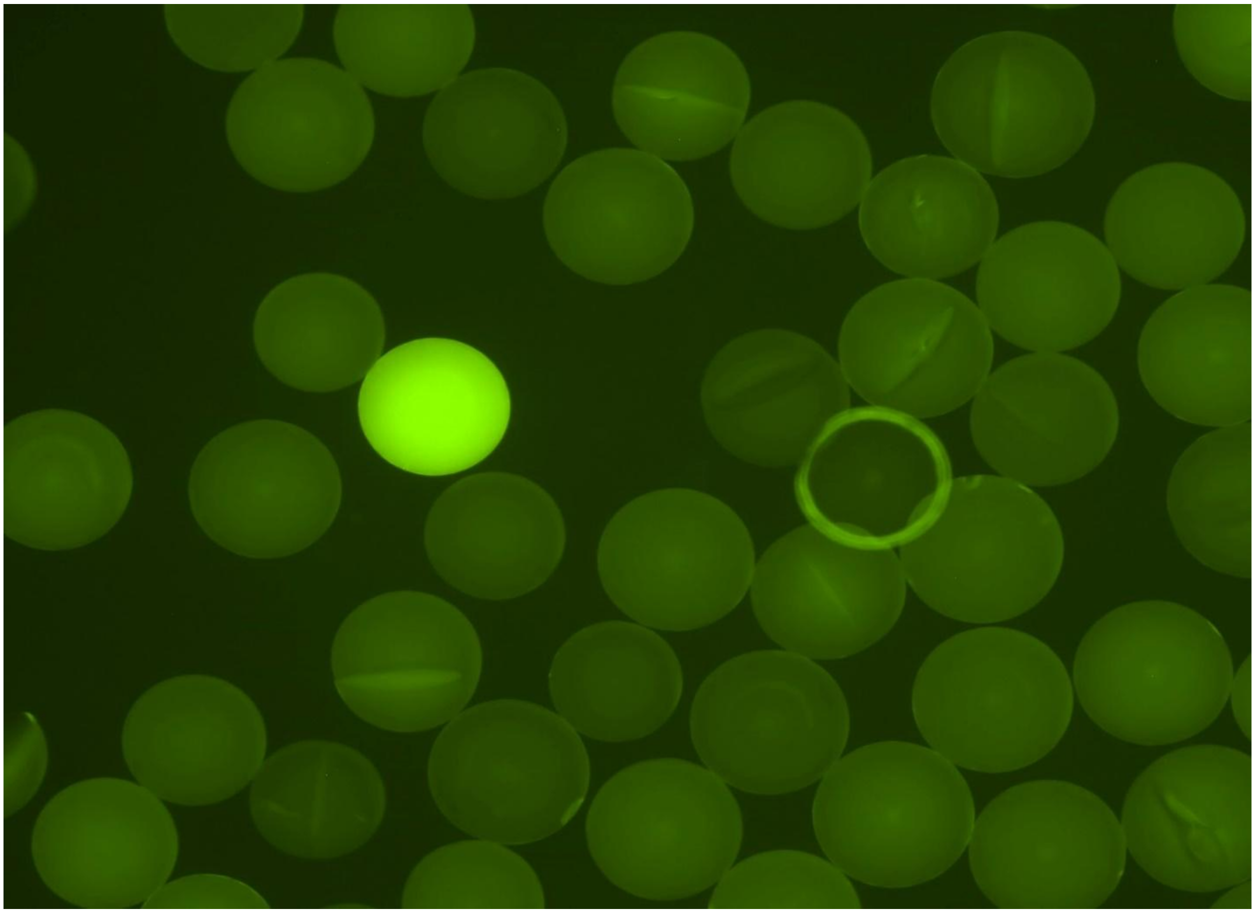

Supplementary Fig. S3. Representative picture of beads for screening. The bright green bead is the positive bead picked up manually.

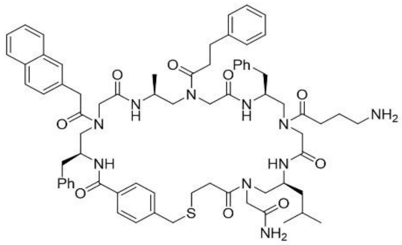

S-1

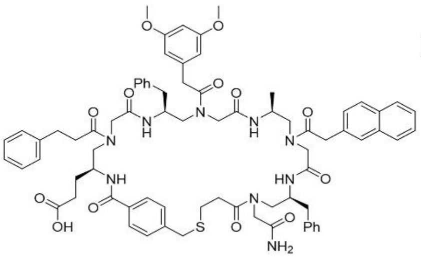

S-2

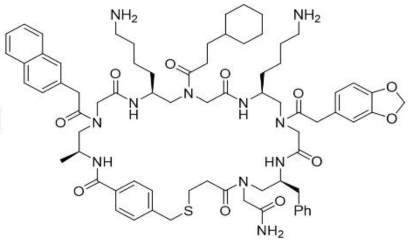

S-3

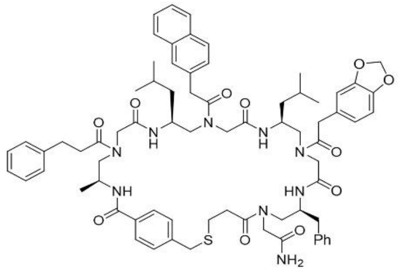

S-4

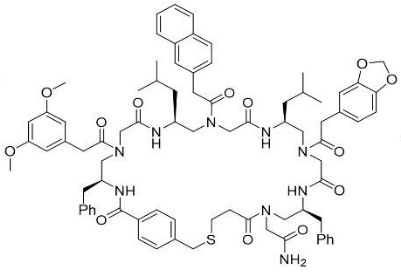

S-5

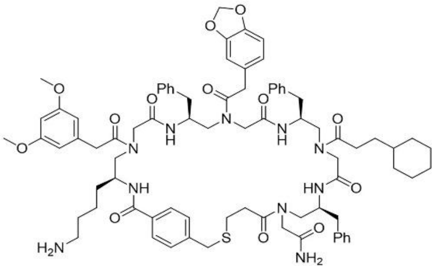

S-6

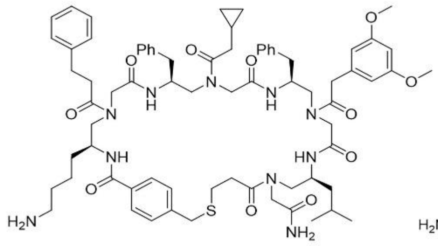

S-7

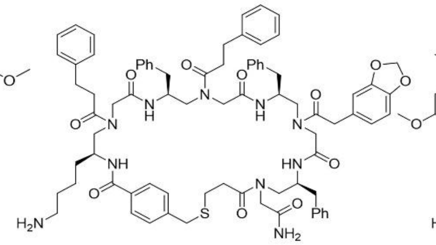

S-8

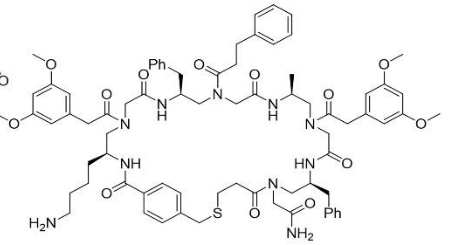

S-9

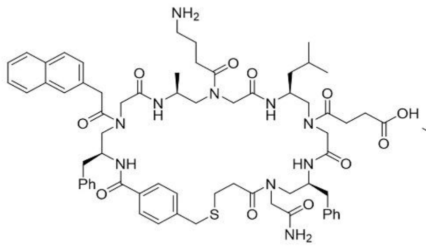

S-10

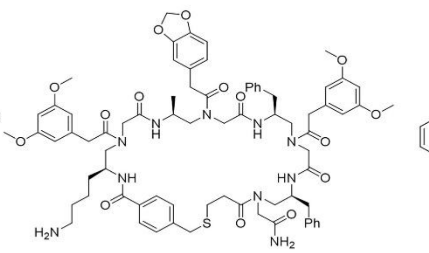

S-11

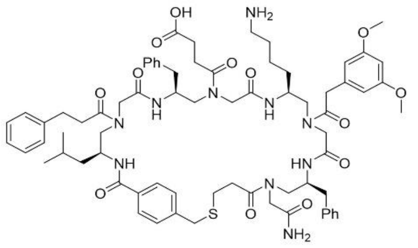

S-12

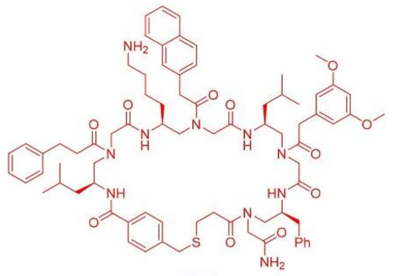

S-13

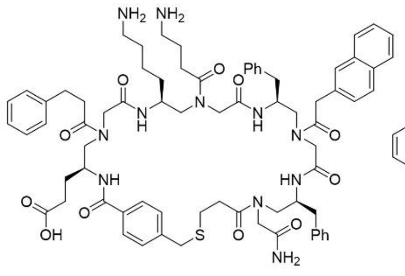

S-14

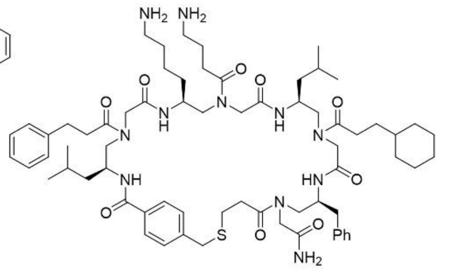

S-15

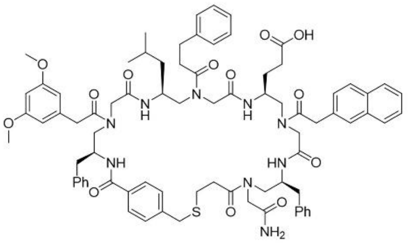

S-16

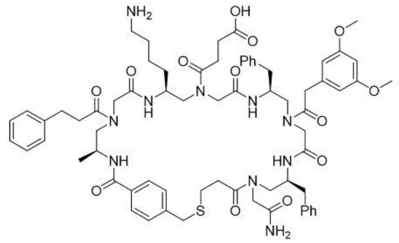

S-17

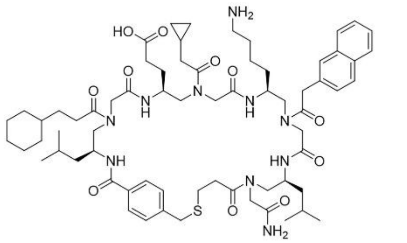

S-18

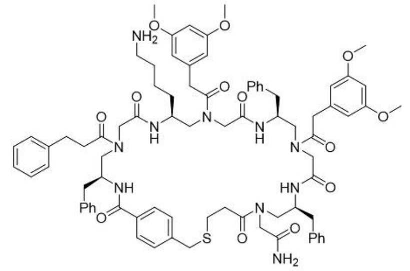

S-19

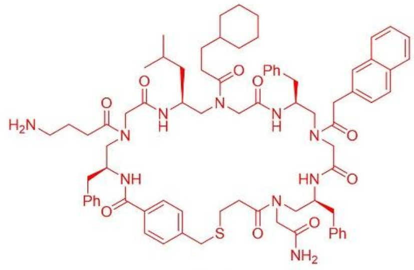

S-20

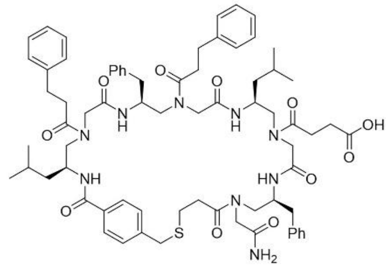

S-21

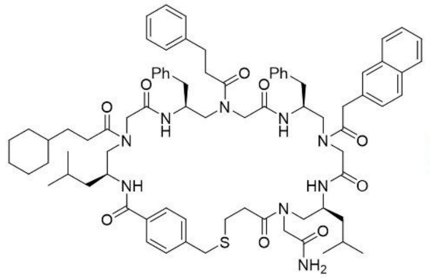

S-22

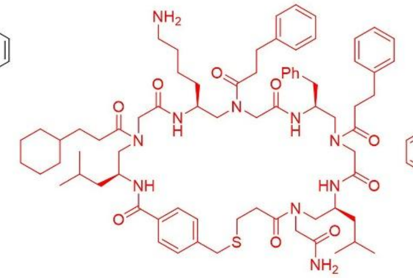

S-23

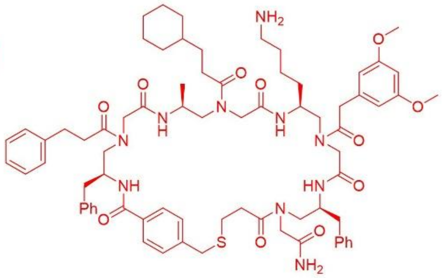

S-24

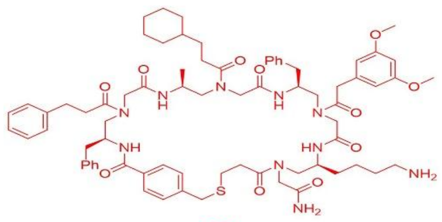

S-25

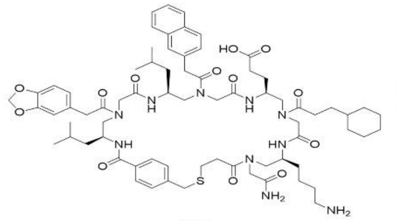

S-26

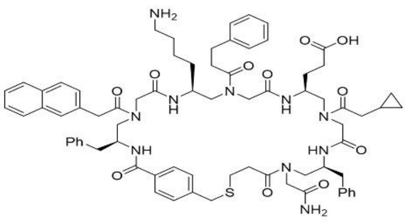

S-27

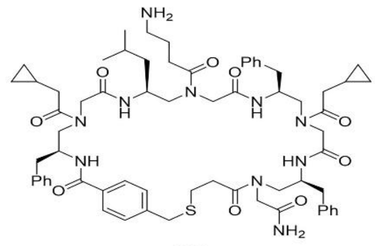

S-28

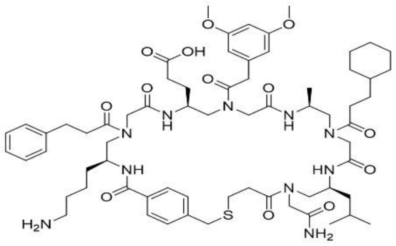

S-29

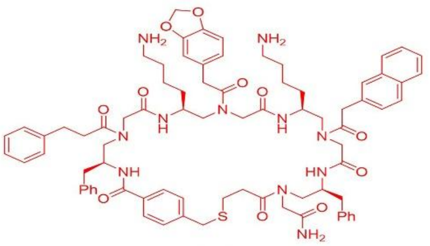

S-30

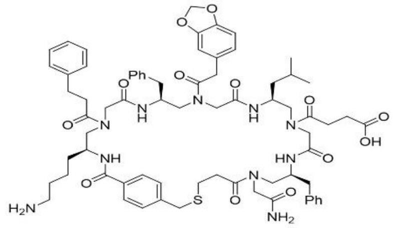

S-31

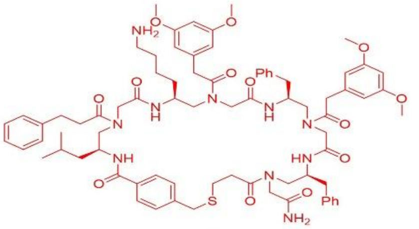

S-32

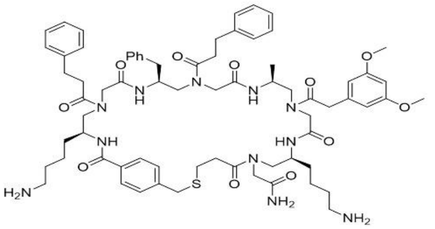

S-33

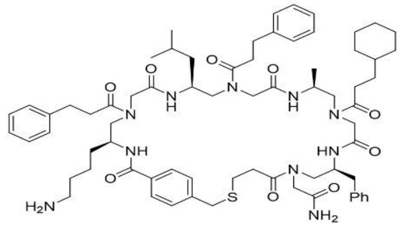

S-37

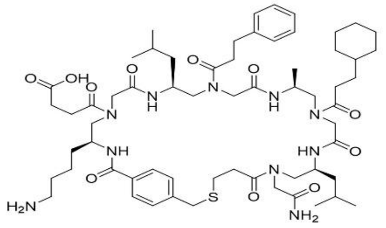

S-38

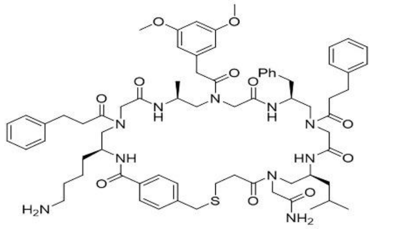

S-39

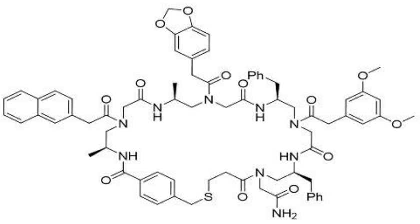

S-40

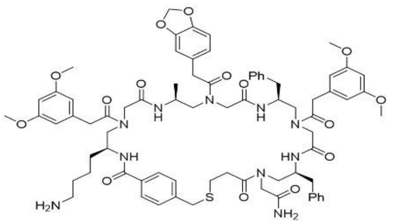

S-41

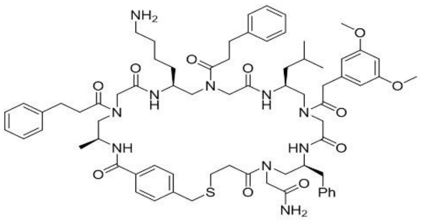

S-42

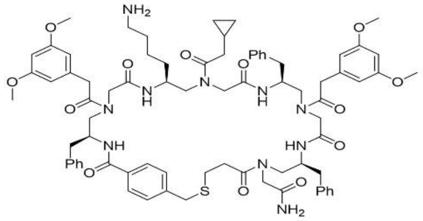

S-43

Supplementary Fig. S4. Chemical structures of cyclic γ-AApeptide compounds S-1 to S-43. Structures highlighted in red are the seven lead compounds shown in Fig. 1c.

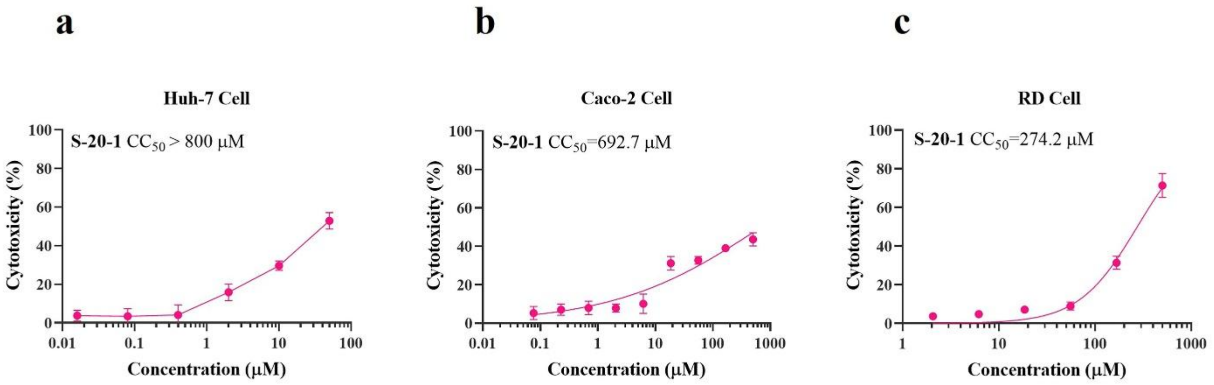

Supplementary Fig. S5. Cytotoxicity of S-20-1 on Huh-7 (a), Caco-2 (b) and RD cells (c).

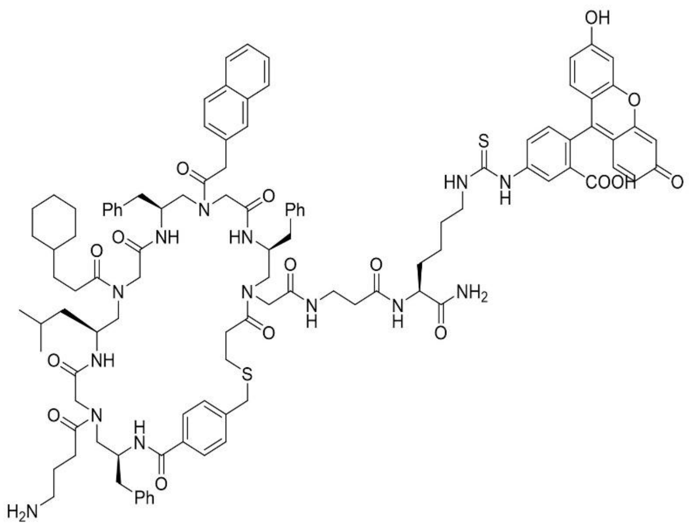

FITC-S-20

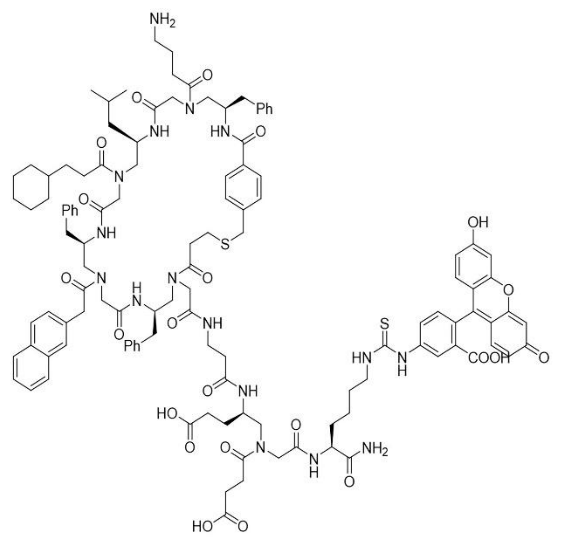

FITC-S-20-1

Supplementary Fig. S6. Chemical structures of FITC-labeled cyclic γ-AApeptides S-20 and S-20-1.

a. HPLC analytic trace of **S-20**.

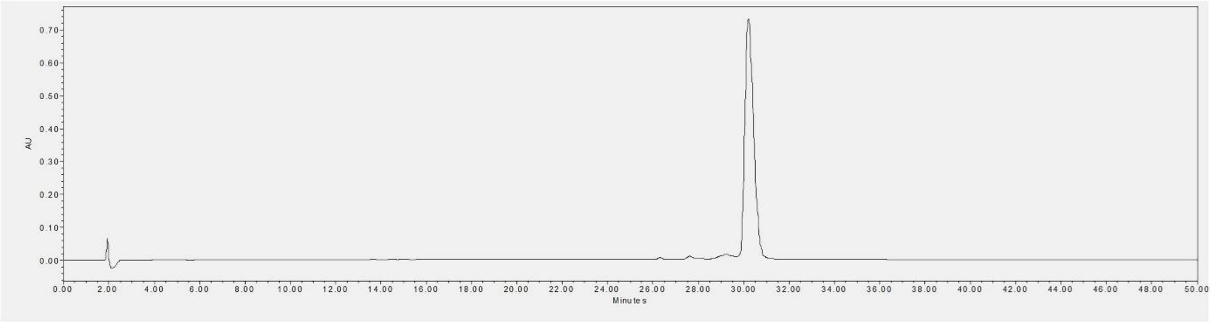

b. HPLC analytic trace of **S-20-1**.

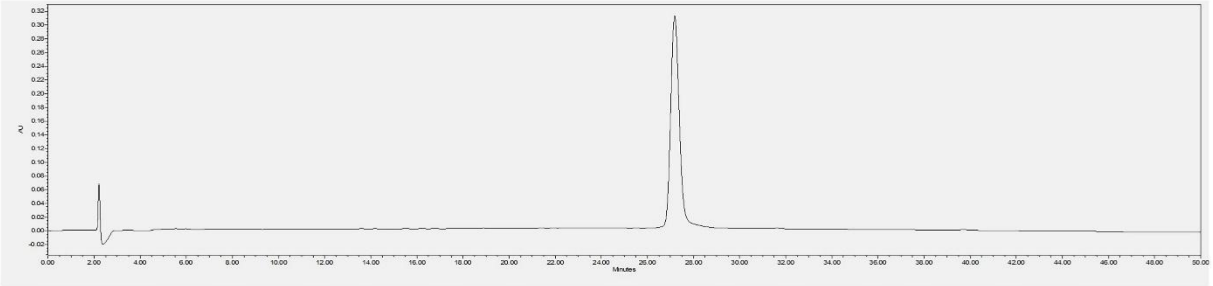

c. HPLC analytic trace of **FITC-S-20**.

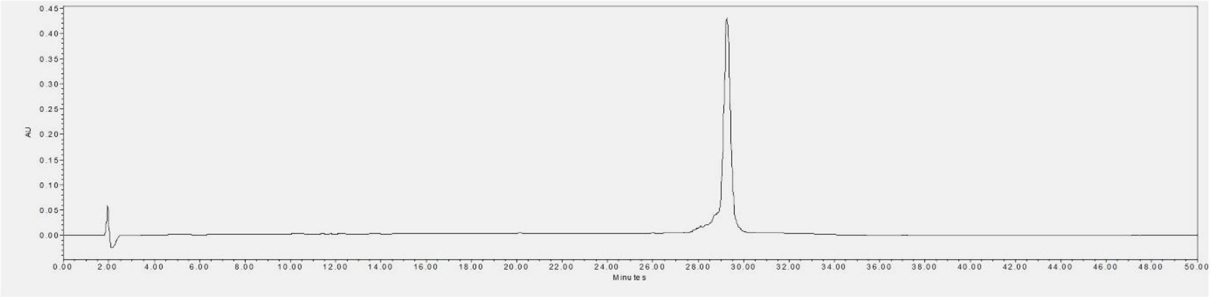

d. HPLC analytic trace of **FITC-S-20-1**.

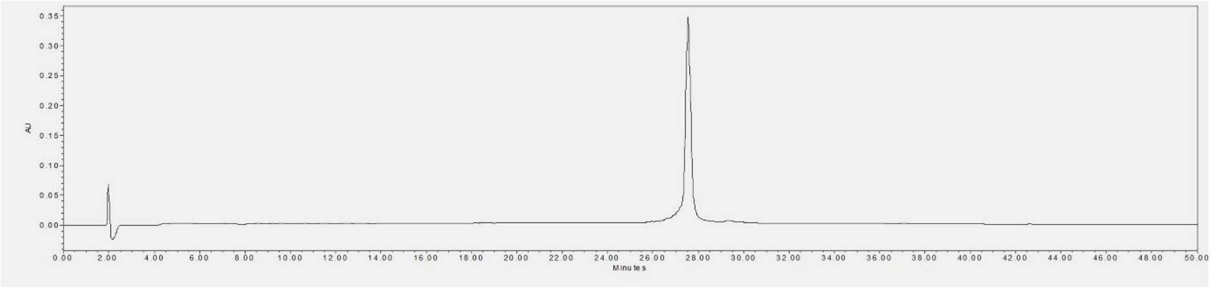

Supplementary Fig. S7. HPLC analytic trace of S-20 (a), S-20-1 (b), FITC-S-20 (c) and FITC-S-20-1 (d).

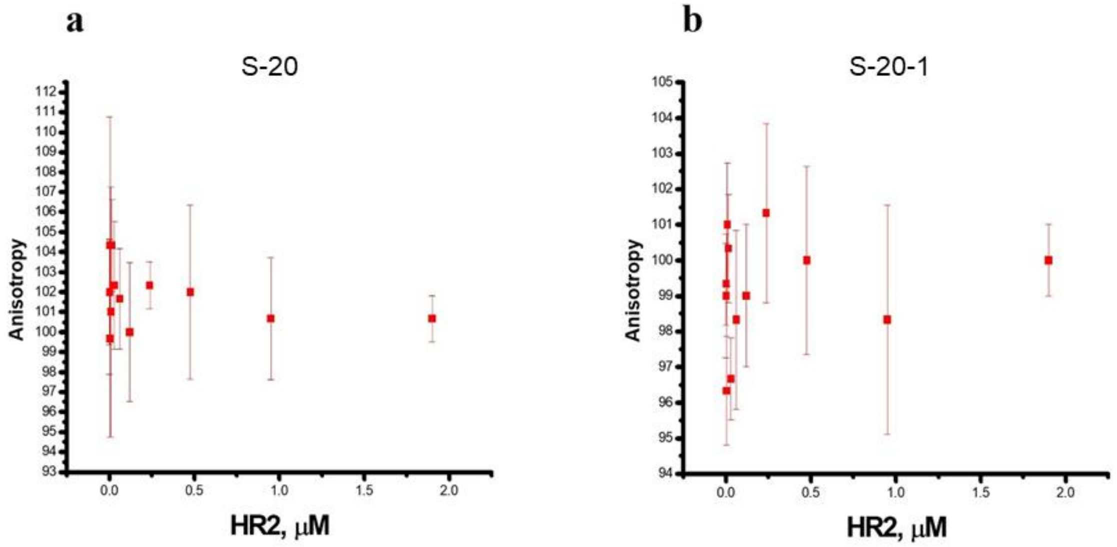

Supplementary Fig. S8. Binding affinity of **S-20** (a) and **S-20-1** (b) to HR2 protein measured by fluorescence polarization (FP) assay.

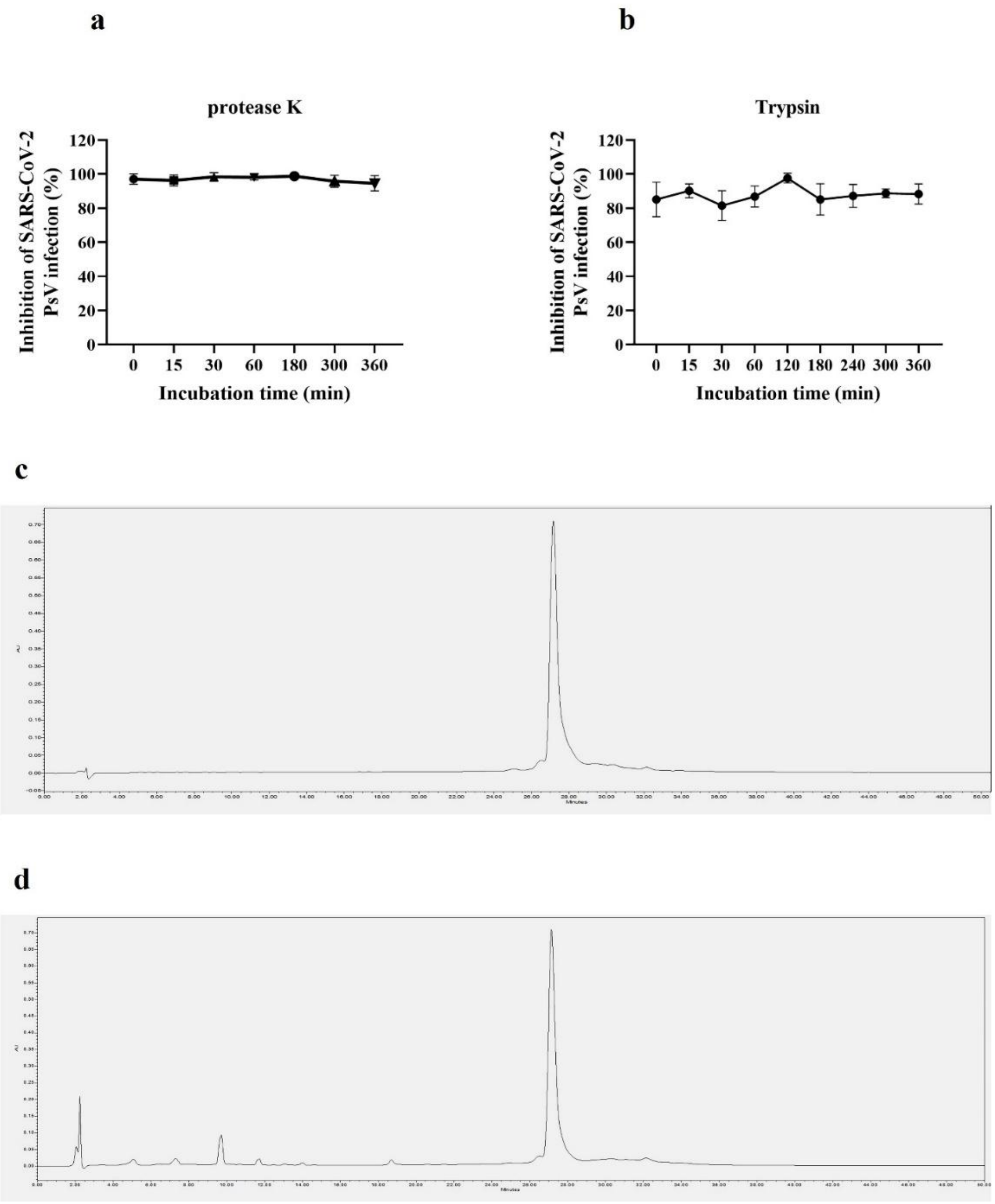

Supplementary Fig. S9. Evaluation of the stability of sequences in various proteolytic enzymes. Metabolic stability of **S-20-1** in proteinase K (a) and trypsin (b). Analytic HPLC traces of **S-20-1** before (c) and after (d) incubation with Pronase (0.1 mg/ml).

a

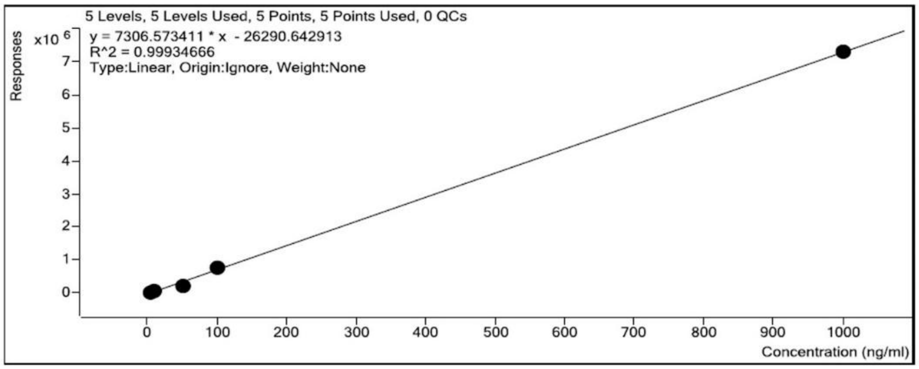

b

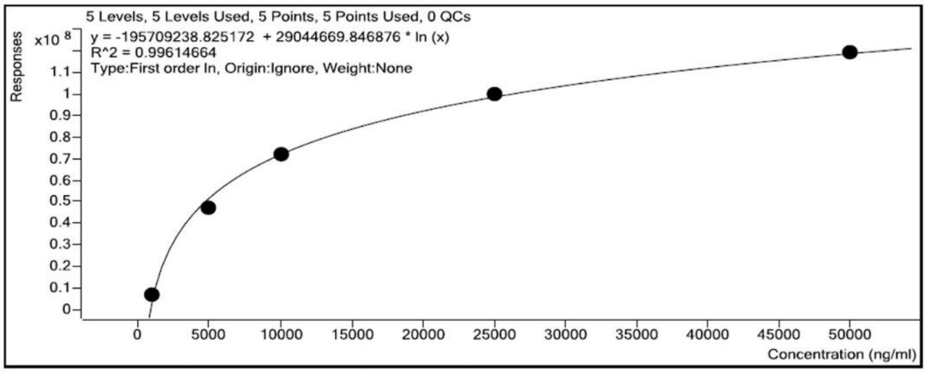

c

X	Group A		Group B	
Time (h)	OP		IP	
X	Mean	SD	Mean	SD
0.17	894.88	227.86	5261.581467	264.6199626
0.33	1613.15	478.03	16540.285730	2044.0870140
0.50	534.56	85.86	30171.880400	4500.6254660
1.00	914.99	200.92	16705.920000	4206.7130000
2.00	1033.63	86.51	120637.086900	7489.0678730
4.00	2694.12	672.74	63311.230000	15205.3200000
8.00	899.08	302.90	51428.262800	9625.8350760
24.00	207.72	43.95	6583.937000	1085.7360000
48.00	159.03	23.49	7140.970267	992.6039837

Supplementary Fig. S10. Standard calibration curve for low concentration (5 ng/ml to 1000 ng/ml) (a) and high concentration (1 μg/ml to 50 μg/ml) (b). The concentration of **S-20-1** at different time points (c).

Supplementary Table S1. Inhibitory activity, cytotoxicity, and selective index (SI) of the 7 lead compounds

Compound	IC_{50} (μM)	CC_{50} (μM)	SI	Compound	IC_{50} (μM)	CC_{50} (μM)	SI
**S-13**	5.02	25.5	5.08	**S-25**	3.51	6.52	1.86
**S-20**	2.93	41.94	14.31	**S-30**	4.44	7.16	1.61
**S-23**	2.45	12.75	5.20	**S-32**	12.18	41.95	3.44
**S-24**	4.03	8.54	2.12				

Note: The inhibitory activity and cytotoxicity of the compounds were tested using Huh-7 cells**.**

Supplementary Table S2. Inhibitory activity, cytotoxicity, and selective index (SI) of the 4 selected compounds

Compound	IC50 (μM)	CC50 (μM)	SI
**S-20-1**	0.8	> 800	> 1000
**S-23-1**	1.83	174.7	95.46
**S-24-1**	1.05	> 800	> 761.9
**S-25-1**	1.66	> 800	> 481.93

Note: The inhibitory activity and cytotoxicity of the compounds were tested using Huh-7 cells.

Supplementary Table S3. HRMS of all compounds including FITC labeled compounds

Compound	HRMS (ESI) ($[M+2H]^{2+}$) Calcd	HRMS (ESI) ($[M+2H]^{2+}$) Found	Molecular weight
**S-20**	670.3692	670.3697	1,338.7239
**S-20-1**	841.9382	841.9393	1,681.8618
**FITC-S-20**	964.9549	964.9544	1,926.8918
**FITC-S-20-1**	1,101.0053	1,101.0040	2,200.6440

MATERIALS AND METHODS

1. Screening of One-Bead-Two-Compound library

Prescreening

All TentaGel beads were left to swell in DMF for 1 h, washed with Tris buffer three times, and equilibrated in Tris buffer overnight. After that, the beads were incubated with blocking buffer (1% BSA in Tris buffer with 1000× excess of Escherichia coli lysate) for 1 h. After thorough washing with Tris buffer, beads were incubated with 6×-His-Tag Monoclonal Antibody (HIS. H8) and Dylight 488 (1: 1000 dilution) for 2 h at room temperature. Beads were washed with Tris buffer, and any beads emitting green fluorescence were picked up manually under microscopy and excluded from the next screening. The remaining beads were washed with Tris buffer and denatured by 8 M guandine·HCl for 1 h, followed by washing with DI water (5×), Tris buffer (5×) and DMF (5×). Finally, beads were incubated with DMF for 1 h and then equilibrated with Tris buffer overnight.

Screening

Beads were incubated with blocking buffer (1% BSA in Tris buffer with 1000× excess of Escherichia coli lysate) for 1 h at room temperature. After washing with Tris buffer four times, beads were incubated with SARS-CoV-2 Spike Protein S1/S2 (aa11-1208) and His Tag Recombinant Protein at the concentration of 50 nM for 4 h with 1% BSA in Tris buffer and 1000× excess of Escherichia coli lysate. After thoroughly washing with Tris buffer, beads were incubated with 6×-His Tag Monoclonal Antibody (HIS. H8) and Dylight 488 (1: 1000 dilution)

for 2 h at room temperature. Next, beads were washed with Tris buffer four times and transferred into a six-well plate to be screened under a fluorescence microscope. Beads emitting green fluorescence were picked up as the putative hits.

Cleavage and Analysis

Each positive bead was transferred into a 1.5 ml Eppendorf microtube and denatured in 100 μL 8 M guanidine·HCl for 1 h at room temperature. After thoroughly washing with Tris buffer, water, DMF, ACN, in the end, the bead was placed into ACN overnight in each microtube and allowed ACN to evaporate. Beads were cleaved in a 5:4:1 (v/v/v) solution of ACN/glacial acetic acid/H_2O containing cyanogen bromide (CNBr) at a concentration of 50 mg/mL overnight at room temperature. After evaporation, the residue was dissolved in ACN/H_2O (1:1) and analyzed by MALDI-TOF.

Synthesis of FITC-labeled Cyclic γ-AA peptides

FITC-labeled cyclic γ-AA peptides were synthesized following our previous report.[1,2] Briefly, Fmoc-Lys (Dde)-OH was first attached to Rink amide resin. After removing the Fmoc protecting group, the desired building blocks for sequence synthesis were added. Then the γ-AA peptides were cyclized, removing the Dde protecting group and coupling with Fmoc-β-Ala. The removed Fmoc protecting group was reacted with FITC. FITC-labeled cyclic γ-AA peptides were cleaved by 1: 1(v/v) DCM/TFA containing 2% triisopropylsilane and purified by the Waters HPLC system. Detailed structure information can be found in the **Supplementary Fig. S6.**

2. Cell permeability assay

The cell permeability study was conducted following our previous report.[3] Briefly, HeLa cells were plated in confocal dishes and serum-starved overnight. Following that, HeLa cells were treated with 1 μM FITC-labeled **S-20** or **S-20-1**, respectively, for 2 h and then washed with PBS buffer three times. Next, the cells were fixed with MeOH for 5 min at room temperature, followed by washing with PBS three more times. Cells were then incubated with 1 μg/mL DAPI/PBS for 15 min in the absence of light, followed by thoroughly washing with PBS again. Finally, cells were observed by the inverted Nikon fluorescence microscope.

3. Fluorescence polarization assay

50 nM FITC-labeled γ-AApeptides were incubated with protein (0-2 μM) in PBS. Dissociation constants (K_d) were determined by plotting fluorescence anisotropy values as a function of protein concentration, and the plots were fitted to the following equation.

$$y = [FPmin + (FPmax - FPmin)]$$

$$\frac{(K\mathrm{d}+Lst+x)-\sqrt{(K_d + L_{st} + x)^2 - 4L_{st}\times x}}{2L_{st}}$$

L_{st} and x refer to the concentration of the peptide and protein, respectively. The experiments were conducted in triplicate and repeated three times.

4. Molecular docking studies

Molecular docking studies were carried out as previously described.[2] The molecular docking of **S-20** toward RBD and HR1 was carried out using the Schrödinger Glide program. The conformational search of **S-20** was performed using mixed torsional/low-mode sampling as implemented in Schrödinger (2015) with AMBER force field. The RBD (PDB: 6M0J) and HR1 (PDB: 7C53) of SARS-CoV-2 were chosen for docking. After removal of water and redundant small molecules using PyMol, the proteins were prepared using Schrödinger Protein Preparation Wizard with default settings. Grids were generated using the centroid of the interaction surface as the centers for docking. Docking was performed using the Glide module in Schrödinger (2015) with default parameters.

5. PAMPA-BBB assay

Following a previous report,[2] the PAMPA-BBB assay procedure was developed by pION. All liquid handling steps were performed on the TECAN Freedom EVO150 robot and analyzed by Pion PAMPA Evolution software. BBB PAMPA included brain the sink buffer (BSB), lipid solution (BBB-1) and Stirwell^{TM} PAMPA Sandwich plate preloaded with magnetic stirring disks. 4 µL of lipid solution were transferred into the acceptor well to which 200 µL of BSB (pH 7.4) were added. Then, 180 µL of diluted test compounds (50-250 µM in system buffer at pH 7.4 from a 10 mM DMSO solution) were added to the donor wells. The PAMPA sandwich plate was assembled, placed on the Gut-Box^{TM} and stirred with 60 µm Aqueous Boundary Layer (ABL) settings for 1 h incubation. Distribution of compounds in the donor and acceptor buffer (150 µL aliquot) was determined by UV spectra measurement from 250 to 498 nm using the TECAC

Infinite M-1000 Pro microplate reader. Permeability (P_{app}, 10^{-6}cm/s) of each compound was calculated by Pion PAMPA evolution software. The assay was performed in triplicate.

6. PAMPA-GIT assay

PAMPA-GIT assay[2] was also realized by using a method developed by pION. We also used the TECAN Freedom EVO150 robot to perform all liquid handling steps and analyzed the data by pION's PAMPA Evolution software. The pION's GIT PAMPA includes the acceptor sink buffer (ASB), GIT-0 Lipid solution and the Stirwell™ PAMPA sandwich plate preloaded with magnetic disks. Four µL of lipid were transferred in the acceptor well, followed by addition of 200 µL of ASB (pH 7.4). Then, 180 µL of diluted test compound (50-250 µM in system buffer at pH 5.0, 6.2 and 7.4 from a 10 mM DMSO solution) were added to the donor wells. The PAMPA sandwich plate was assembled and placed on the Gur-Box™ and stirred with 40 µm Aqueous Boundary Layer (ABL) settings for 30 min. Distribution of the compounds in the donor and acceptor buffers (150 µL aliquot) was determined by UV spectra measurement from 250 to 498 nm using the TECAN Infinite M-1000 Pro microplate reader. Then the Permeability (P_{app}, 10^{-6}cm.s^{-1}) of each compound was calculated by Pion PAMPA evolution software. The assay was performed in triplicate.

7. Assessment of enzymatic stability of S-20-1

Cyclic γ-AA peptides **S-20-1** (0.1 mg/mL) were incubated with 0.1 mg/mL protease in 100 mM ammonium bicarbonate buffer (pH 7.8) at 37 ^{o}C for 24 h. After that, water and ammonium bicarbonate in the reaction mixtures were removed using speed vacuum. The residues were dissolved in 100 µL H_2O/ACN and analyzed on a Waters analytical HPLC system.

References

1 Huang, B. *et al.* Activation of E6AP/UBE3A-Mediated Protein Ubiquitination and Degradation Pathways by a Cyclic gamma-AA Peptide. *J. Med. Chem.* **65**, 2497-2506 (2022).

2 Zheng, M. *et al.* Discovery of Cyclic Peptidomimetic Ligands Targeting the Extracellular Domain of EGFR. *J. Med. Chem.* **64**, 11219-11228 (2021).

3 Sang, P. *et al.* Inhibition of beta-catenin/B cell lymphoma 9 protein-protein interaction using alpha-helix-mimicking sulfono-gamma-AApeptide inhibitors. *Proc. Natl. Acad. Sci. U. S. A.* **116**, 10757-10762 (2019).
